# Interventions addressing violence against women in health services: An overview of systematic reviews regarding barriers and facilitators to implementation

**DOI:** 10.1002/ijgo.70796

**Published:** 2026-01-05

**Authors:** Odette del Risco Sánchez, Larissa Rodrigues, Erika Zambrano, Fernanda Garanhani Surita

**Affiliations:** ^1^ Department of Obstetrics and Gynecology, School of Medical Sciences Universidade Estadual de Campinas Campinas Brazil; ^2^ School of Nursing Universidade Estadual de Campinas Campinas Brazil

**Keywords:** gender‐based violence, health promotion, intervention, overview, screening, women's health services

## Abstract

**Background:**

Violence against women is a global issue rooted in gender inequities, requiring coordinated responses within the healthcare system. However, both providers and users face significant challenges in effectively implementing interventions to address it.

**Objectives:**

To synthesize and critically discuss, from the perspectives of professionals and users, the barriers and facilitators to implementing interventions addressing violence against women within healthcare services.

**Search Strategy:**

This umbrella review combined the Joanna Briggs Institute's method and the Evidence Synthesis for Health Policy. The search was conducted from databases (PubMed, *Biblioteca Virtual em Saúde* (BVS), PsycInfo, Scopus, Embase, Web of Science, and the Cochrane Library).

**Selection Criteria:**

Systematic reviews were included if they allowed for the analysis of individual, relational, contextual, and cultural factors involved in the interventions.

**Data Collection and Analysis:**

Data extraction focused on study characteristics and findings related to implementation barriers and facilitators. A narrative synthesis was undertaken, enabling a discussion of recommendations for policy, research, and practice.

**Main Results:**

Among the 11 systematic reviews, primary studies from high‐income countries were noted, with a notable emphasis on primary healthcare services. The successful implementation of interventions is shaped by a complex interplay of individual, relational, cultural, and systemic factors. Key determinants influencing implementation included workforce education, providers' and survivors' feelings, healthcare provider attitudes, prevailing cultural norms, and institutional support mechanisms.

**Conclusions:**

The implementation of interventions requires a comprehensive approach that takes into account the complex nuances influencing the success of efforts to address violence against women in healthcare settings.

## 1 INTRODUCTION

Violence against women (VAW) remains a persistent problem globally.^1^ Although VAW is an issue in all societies and groups, showing a high prevalence at the global level, there is considerable variability in its prevalence and patterns across countries and regions. Data from the WHO indicate that the highest rates of lifetime VAW are concentrated in the regions of Oceania, South Asia, and sub‐Saharan Africa, and the lowest rates occur in Europe (16%–23%), Central Asia (18%), East Asia (20%) and Southeast Asia (21%). In North America, Latin America and the Caribbean, the rate is 25% for each region.^2^

Furthermore, violence incurs social and economic costs that affect both individuals and their communities, with estimates suggesting these costs amount to 1.5 trillion dollars, which represents 2% of the global gross domestic product.^3^

Women who have experienced intimate partner violence (IPV) tend to seek healthcare more frequently than women who have not.^4^ Studies highlight an increased use of services such as emergency departments, hospital outpatient clinics, primary care, pharmacies, and specialty services.^5^, ^6^

Nevertheless, the underreporting of victims of violence is a recurrent problem, making it difficult to estimate the actual number of victims and consequently limiting the access of the survivors to adequate care.^7^ This situation calls for the development of strategies aimed at identifying and reducing the barriers survivors face when reporting violence within relationships, as well as addressing gaps in reporting.

Healthcare professionals play an important role in identifying women in situations of violence. There are strategies for the development of actions aimed at preventing and reducing violence within health care services, including routine screening, referrals to specialized services within and outside the healthcare system, first‐line support, among others.^8^, ^9^

Studies highlights that interventions' effectiveness in health care systems vary based on several factors, including the healthcare setting and level, the study population characteristics, methodological issues, and the types of interventions, their contents, and duration.^9^, ^10^, ^11^, ^12^ These interventions are diverse; therefore, robust evidence is needed to support evidence‐informed policymaking and to strengthen health systems.^10^ Therefore, it is important to discuss the barriers faced by users, healthcare professionals, and managers in implementing these initiatives.

To implement effective interventions, inherent challenges exist within professional practice and healthcare service management, particularly concerning interventions addressing sensitive issues such as violence.^9^, ^13^

In this context, identifying barriers and facilitators for the implementation of these interventions can contribute to the improvement of the interventions themselves, as well as the implementation and evaluation processes, thereby expanding opportunities for action and the replication of successful practices within healthcare settings.

Through this review, we aimed to synthesize and critically discuss the barriers and facilitators of interventions within this scope, with the purpose of offering applicable recommendations, emphasizing the elements that, from the perspectives of both professionals and users, influence the implementation and effectiveness of VAW interventions.

### 1.1 Aims of the review

- To explore the barriers and facilitators involved in implementing interventions addressing VAW within health services, from the perspectives of healthcare providers and users;
- To categorize the factors that hinder or support the implementation of such interventions in health service settings;
- To identify lessons learned from the literature regarding best practices for implementing these interventions.

### 1.2 Review question

What are the enablers and barriers that influence the implementation of interventions addressing VAW within health services, from the perspectives of healthcare providers and users?

## 2 METHODS

This review was conducted by combining the Joanna Briggs Institute (JBI) method for umbrella reviews^14^ and the Evidence Synthesis for Health Policy.^15^, ^16^ We compiled the best available evidence to understand the patterns of the problem; describe the possible impacts of the main interventions and provide information and considerations about potential barriers to implementing the options and strategies for addressing those barriers.^16^ The protocol was registered in Open Science Framework (DOI https://doi.org/10.17605/OSF.IO/548MV).

### 2.1 Eligibility criteria

Regarding the methodological design of the studies, we only included systematic reviews, that address perceptions of strategies for the prevention of domestic and family VAW and/or adolescents at any level of care; approached of healthcare professionals, managers and/or healthcare services users; and analyze individual, relational, contextual, organizational, and cultural factors involved in the interventions.

Systematic reviews that do not explore barriers and facilitators of interventions on VAW and/or adolescents within or including health settings, do not target women and adolescents who are victims and/or at risk of violence, health managers and/or professionals, are not related to the target population's perception of domestic and family violence prevention strategies, or whose outcomes were not accessible, were excluded.

### 2.2 Search strategy

Searches were conducted in National Library of Medicine (NLM) (PubMed), *Biblioteca Virtual em Saúde* (BVS), American Psychological Association (APA) PsycInfo, Scopus, Excerpta Medica dataBASE (Embase), Web of Science, and the Cochrane Library in January 2025. The search strategy followed the PICO strategy. No date limits were applied in the search strategy. An initial search of PubMed was carried out to perform a preliminary analysis of the keywords found in the titles and abstracts of the studies, as well as the index terms used to describe them (Table S1).

To ensure optimal retrieval, the search strategy was adjusted to align with the indexing of each database without temporal restrictions. The search strategy was developed collaboratively by a health sciences librarian and researchers with expertise in conducting reviews. Search terms were defined using DeCS/MeSH (Health Sciences Descriptors/Medical Subject Headings) and supplemented with free‐text terms to ensure a comprehensive and exhaustive search.

Hand searching strategies were also employed in order to achieve greater precision in the retrieved studies.

### 2.3 Screening and selection

The selection criteria were tested through a pilot test involving a sample of 25 titles/abstracts, evaluated by the team based on the eligibility criteria, the research question, and the elements outlined in the initial protocol. All the papers identified were imported to the Rayyan Qatar Computing Research Institute software (Rayyan QRI. https://www.rayyan.ai/) and any duplicates were removed. Two researchers conducted an independent and blind screening based on titles and abstracts. Subsequently, the papers were fully screened, and any conflicts were discussed to reach consensus.

### 2.4 Data extraction

A modified version of the standardized framework from the Guideline for Evidence Synthesis for Health Policy was employed.^16^ The following information was systematically extracted: author(s) and year of publication, authors' country, title, type of study, study objectives, type of interventions addressed, type of violence, healthcare setting, target population, proportion of studies conducted in low‐ and middle‐income countries, benefits of the interventions, potential harms of the interventions, individual, relational, and contextual factors influencing the implementation of interventions and recommendations.

All selected studies were exported to a Microsoft Excel (Windows) database. To ensure consistency and reliability in the data extraction process, one reviewer performed the initial extraction, which was independently verified by a second reviewer. The data extraction form was collaboratively reviewed, refined, and validated by the research team.

### 2.5 Assessment of methodological quality

Two authors independently performed the methodological quality assessment of the reviews using the JBI Critical Appraisal Checklist for Systematic Reviews and Research Syntheses.^14^ This tool includes 11 questions that evaluate the review methodology, search strategy, and possible bias based on four possible responses (Yes/No/Unclear/Not applicable). While no studies were excluded due to quality, their methodological rigor was considered in the analysis, and potential impacts on the results were discussed by the team. Extracted data were organized using a synthesis matrix to facilitate comparison.

### 2.6 Data synthesis

A narrative synthesis was then undertaken to identify, describe, and summarize the body of evidence. The research team analyzed and discussed best practices based on the characteristics of the interventions and the outcomes reported in the studies from the perspectives of the target public using “building blocks of health systems” proposed by WHO as framework.^17^

Nvivo 14 software (QSR International, MA, USA) supports the procedures for organizing and analyzing articles, including coding and the development of categories that will enable the synthesis of the main barriers and facilitators of interventions.

## 3 RESULTS

**FIGURE 1:**
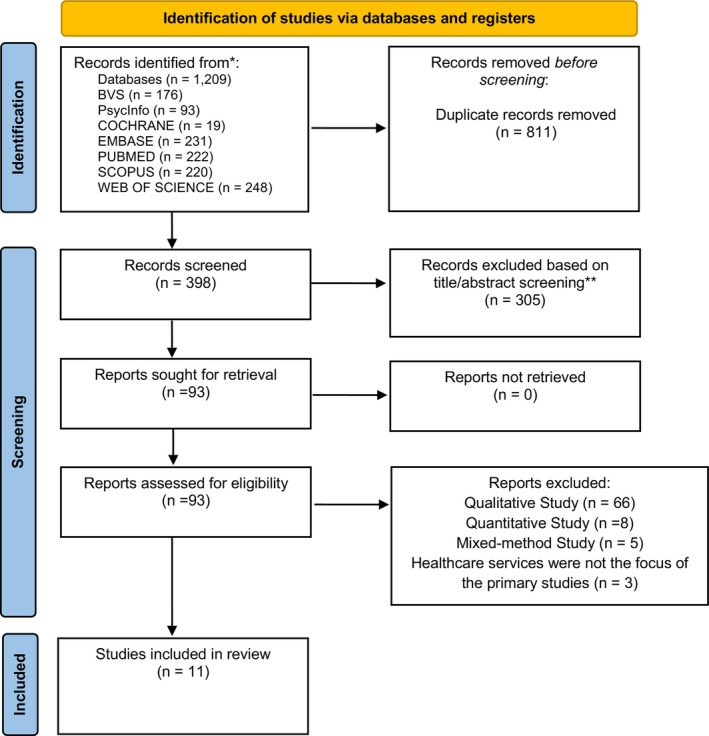
PRISMA flow chart.

In total, 1209 articles were retrieved, and 811 duplicates were removed. A total of 398 articles were screened; 93 were retrieved for full‐text reading, and 11 were kept for analysis. Figure 1 shows details of the selection process.

### 3.1 Characteristics of included studies

A detailed description of the included systematic reviews is presented in Table 1. The reviews were published between 2017 and 2024, all in English. The reviews included a total of 324 studies, ranging from eight to 47 primary studies and cover countries from all continents; however, high‐income countries were more frequently represented among the primary studies included in the reviews.

**TABLE 1:** Characteristics of the studies included.

Author(s)/year/country	Objectives	Settings	Participants of the primary studies	Number of primary studies	Countries and proportion of studies conducted in low‐ and middle‐income countries
Saletti‐Cuesta et al. (2018)^25^ Argentina and Spain	To review and synthesize qualitative studies exploring opinions and experiences of PCPs regarding VAW	PHC	Health professionals (i.e., nurses, physicians, psychologist and social workers, community health agents, gynecologists, midwives, general practition, rs, obstetricians, pediatricians) (primary studies included around 1500 providers)	46	High‐income countries: Canada *n* = 3, USA *n* = 2, UK *n* = 1, Germany *n* = 1, Scotland *n* = 3, Australia *n* = 2 and Greece *n* = 1 Low‐ and middle‐income countries: Brazil *n* = 29, Sri lanka *n* = 1, Serbia *n* = 1, Chile *n* = 1, and Lebanon *n* = 1 33/46
Bundock et al. (2020)^18^ UK	To systematically identify and summarize empirical work examining adolescent victims' help‐seeking behaviors and intentions in relation to their own experience of ADV	Health‐care clinic, urban adolescent center, clinics providing confidential services to adolescents, social service agency, and family planning service	Adolescents aged 10–24 years (*n* = 22 372 primary studies included from 11 to 11 570 participants)	19	High income countries: USA *n* = 18 and New Zealand *n* = 1 0/19
Duchesne et al. (2023)^19^ Canada	To summarize the existing evidence regarding (1) ED care experiences of patients with a history of IPV and (2) experiences of ED providers interacting with them	ED	Patient and provider in emergency care (primary studies included from 1 to 1484 participants)	41	High income: Turkey *n* = 1, UK *n* = 5, Canada *n* = 4, USA *n* = 11, New Zealand *n* = 3, Finland *n* = 2, Sweden *n* = 1, Ireland *n* = 1, Israel *n* = 1, Japan *n* = 1, Netherlands *n* = 1, Australia *n* = 6 Low‐ and middle‐income countries: China *n* = 1, Malaysia *n* = 1, Kenya *n* = 2, South Africa *n* = 1 5/41
Hulley et al. (2023)^23^ UK	To explore barriers to help‐seeking as experienced by Black, Asian, minority ethnic and immigrant women with experience of intimate partner violence	Healthcare services, shelters, social service agencies and other community services	Black, Asian, minority ethnic and immigrant women with experience of IPV Around 921 Black, Asian, minority ethnic women, of whom approximately 630 were immigrants (primary studies included from 6 to 86 participants)	47	High‐income countries: UK *n* = 4, USA *n* = 34, Australia *n* = 2, Norway *n* = 1, Canada *n* = 1, Taiwan *n* = 1, Hong Kong *n* = 3, UK‐ Sweden *n* = 1 0/47
Ravi et al. (2022)^20^ USA	To identify key factors that facilitate survivors' formal help‐seeking	Public institutions, medical and health clinics, legal/judicial services, and community agencies	Survivors of IPV seeking health services (adults aged 18 or older) (primary studies included from 13 to 163 participants)	24	High income country: USA *n* = 24 0/24
Lu et al. (2024)^22^ Australia	To determine the barriers that face obstetricians/ gynecologists regarding enquiry of IPV in antenatal outpatient settings	Antenatal outpatient care	Doctors providing antenatal outpatient care (primary studies included from 21 to 993 participants)	11	High income countries: USA *n* = 4, Canada *n* = 2, Belgium *n* = 1, New Zealand *n* = 1, France *n* = 1 and Pakistan *n* = 2 0/11
Femi‐Ajao et al. (2020)^27^ UK	To summarize evidence from qualitative research on domestic violence and abuse among women from BME groups in the UK. The objectives of the review were to explore the (1) barriers to disclosure, (2) facilitators of help‐seeking and (3) self‐perceived impacts of domestic violence	Domestic violence services, refuges, social service	BME women (18 years and above) with lived experience of domestic violence and abuse in UK (*n* = 83 participants, 58 participants were from South Asian BME (Indian, Pakistani and Bangladeshi) groups, three African participants, 3 African‐Caribbean participants, 5 Jewish and 5 Irish participants, respectively)	8	High income country: UK *n* = 8 0/8
Colombini et al. (2017)^21^ UK	To identify and analyze barriers and facilitators to integrated health sector responses to IPV in LMICs	ANC, PHC, and ED	Adult women survivors of IPV in healthcare settings from LMICs	11	Caribbean and Latin America Brazil, Dominican Republic, Peru and Venezuela *n* = 1 Peru *n* = 1 Asia: Hong Kong *n* = 1, Malaysia *n* = 1, Bangladesh *n* = 1 Africa: South Africa *n* = 5 and Kenya *n* = 1 11/11
Brown et al. (2022)^24^ Australia and UK	To explore the experiences of survivors of sexual abuse and violence who received interventions to support them and improve their health and well‐being, as well as experiences of their family members and the professionals who delivered such interventions	Community mental health agency, healthcare setting, inpatient setting, outpatient setting/clinic, trauma outpatient clinic, sexual abuses crisis centers and programs	Survivors of sexual abuse and violence, professionals who took part in interventions that supported survivors of sexual abuse and violence, or family members who supported survivors who completed these interventions *N* = 292 survivors, *n* = 19 survivors' family members or partners and *n* = 60 intervention facilitators	37	High‐income countries: Australia (*n* = 1), Canada (*n* = 5), Iceland (*n* = 1), Ireland (*n* = 1), Norway (*n* = 1), UK (*n* = 5) and USA (*n* = 17) Middle‐income countries: Chile (*n* = 2) and South Africa (*n* = 2) Low‐income countries: Nicaragua (*n* = 1) and the Philippines (*n* = 1) 6/37
Sultana et al. (2023)^28^ UK	To examine the barriers and facilitators of help‐seeking behaviors in South Asian women living in high‐income countries who have experienced DV to inform practice, understand the limits of the evidence, and identify research gaps	Community organizations, researcher's networks, refugee camps and mosques	South Asian ethnic communities in high‐income countries and services providers (primary studies included from 3 to 88 women aged 18 or above living in high countries)	35	High‐income countries: USA *n* = 15, UK *n* = 8, Hong Kong *n* = 2, Sweden *n* = 1, Canada *n* = 9, Norway *n* = 1 Low‐income countries included in multicountry studies: India and USA *n* = 1 Pakistan and UK *n* = 1 2/35
Heron & Eisma (2021)^26^ USA and The Netherlands	To provide an updated synthesis of qualitative research identifying barriers and facilitators, advice, and positive and negative outcomes of adult victims' disclosure of domestic violence to HCPs	DV and women's services, maternity/antenatal clinics, print media, ED/hospitals, general practitioner's offices, and mental health services	Domestic violence survivors *N* = 783 domestic violence survivors (781 females)	34	High‐income countries: USA *n* = 16, Australia *n* = 6, UK *n* = 5, the Netherlands *n* = 1, Canada *n* = 1 and Scotland *n* = 1 Low‐ and middle‐income: Nepal *n* = 1, Malaysia *n* = 1, Jordan *n* = 1, Mexico *n* = 1 4/34

Abbreviations: ANC, antenatal care; BME, Black and minority ethnic; DV, domestic violence; ED, emergency department; HCPs, healthcare professionals; IPV, intimate partner violence; LMICs, low‐ and middle‐income countries; PCPs; primary care providers; PHC, primary health care.

Regarding methods of studies included in the reviews, we found that qualitative studies were included in all the systematic reviews. From 11 reviews, five also incorporated quantitative studies, most of which were cross‐sectional studies,^18^, ^19^, ^20^, ^21^, ^22^ and five included mixed‐methods approaches.^18^, ^19^, ^20^, ^23^, ^24^

About the healthcare context, it was observed that the primary studies were conducted in primary health care settings (*n* = 51)^21^, ^25^; emergency departments (*n* = 45),^19^, ^21^, ^26^ antenatal care services (*n* = 22),^21^, ^22^, ^26^ specialized services for violence survivors (*n* = 24),^24^, ^26^, ^27^ services providing care for adolescents (*n* = 19),^18^ and family planning services (*n* = 1).^18^ Among the four reviews, diverse agencies and community services were observed, including community organizations, refugee camps, shelters, legal/judicial, and social service agencies.^20^, ^23^, ^24^, ^28^

The papers included healthcare providers perspectives,^24^, ^25^ adolescents survivors of dating violence,^18^ adult women survivor of violence that help‐seeking in healthcare settings and/or take part of VAW interventions,^19^, ^20^, ^21^, ^24^ pregnant women,^22^ historically oppressed groups like Black, Asian, minority ethnic and immigrant women.^23^, ^26^, ^28^

The forms of violence addressed in the studies included forms of family and domestic violence, and IPV, including psychological, physical, sexual, financial, and emotional abuse. Despite being less represented, evidence about adolescents dating violence were also addressed.^18^

Most of the reviews discuss barriers and facilitators influencing help‐seeking behavior among survivors of violence.^18^, ^20^, ^23^, ^27^ Additionally, the studies address challenges related to providing emergency care to survivors,^19^ as well as difficulties in screening and/or supporting survivors,^21^, ^22^, ^25^ and implementing psychosocial interventions.^24^

### 3.2 Quality appraisal

The results of the quality assessment are presented in Table S2. Overall, the reviews indicate relatively high quality in research design and conduct, contributing to discussions on implications for clinical practice, research, and policy. The risk of bias in the primary studies is less frequently explored in the reviews, as most of the evidence comes from systematic reviews of qualitative studies.

### 3.3 Thematic analysis

Enablers and barriers that influence the implementation of interventions were mentioned in the systematic reviews. Main themes were identified from the analysis that were grouped in the following thematic themes and subthemes.

### 3.4 Influence of individual factors of providers and survivors on the VAW interventions

#### 3.4.1 Barriers

**FIGURE 2:**
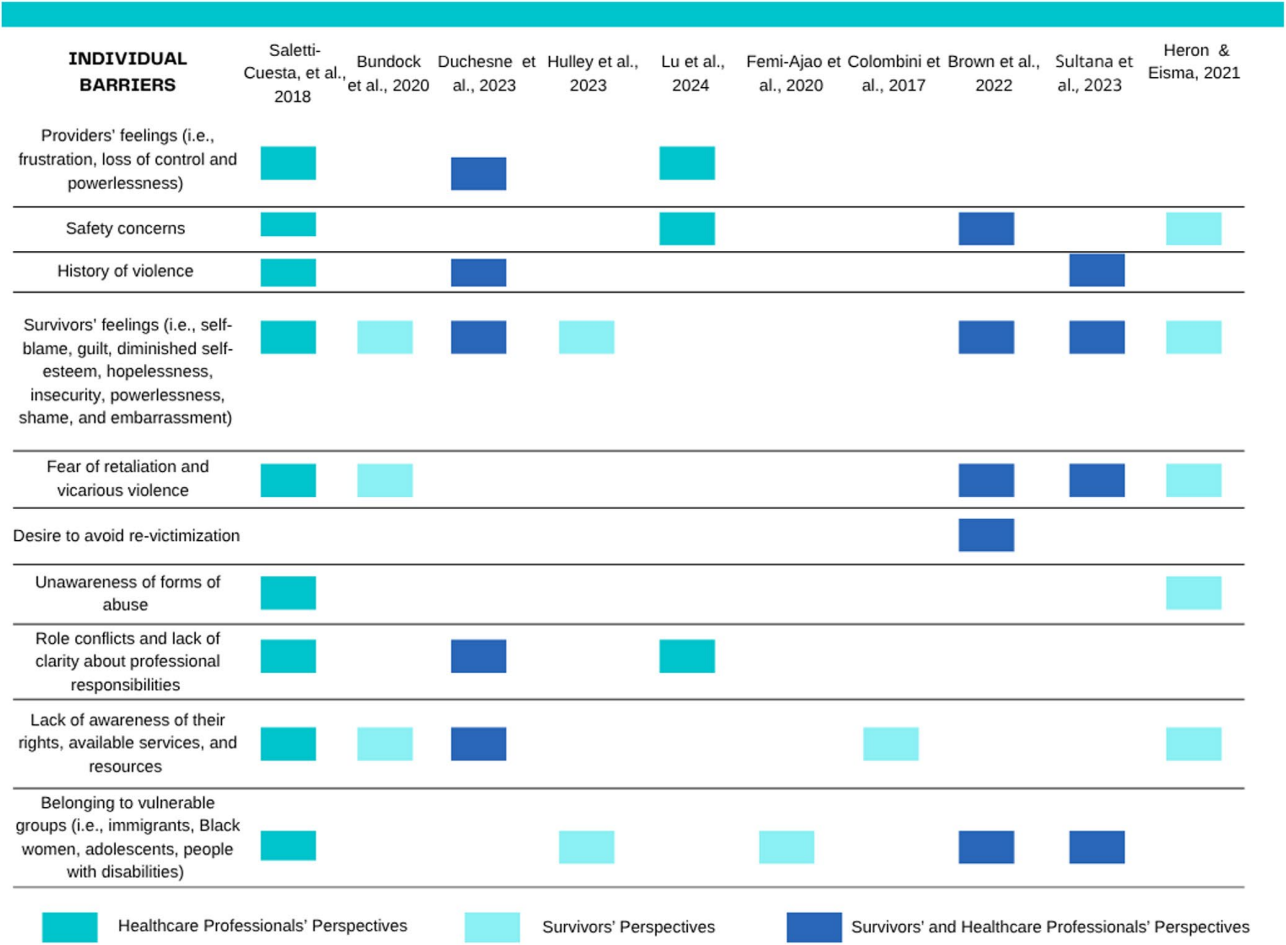
Individual barriers described in the studies.

Figure 2 shows individual barriers from the perspective of providers and VAW survivors. Some evidence shows how providers' feelings such as managing feelings of frustration, loss of control and powerlessness can undermine professionals' self‐confidence and willingness to address VAW as part of the routine.^22^, ^25^ These feelings of frustration tend to be present even when the survivors decline police intervention.^19^

Providers expressed fear of repercussions for their physical and emotional safety, reporting concerns not only for themselves but also for their families, particularly when interventions involved legal procedures.^22^, ^25^

The provider's own history of violence could influence their attitudes to confront this issue, making it difficult to balance their experiences with their professional role.^19^, ^25^ Similarly, a history of violence affects women, and those exposed to domestic violence during childhood may face challenges in coping with these experiences in adulthood.^28^

The perception that the professional's role was confined to health‐related matters limited their ability to address this topic, as it was seen as a social issue, avoiding opening a “pandora's box.”^19^, ^21^, ^22^

Barriers such as emotional vulnerability, social isolation, internalized gender norms, fear of stigma may prevent adult women from seeking help.^25^ Survivors frequently experience a complex mix of emotional responses such as fear of consequences to disclose the abuse, self‐blame, guilt, diminished self‐esteem, hopelessness, insecurity, powerlessness, shame, and embarrassment.^26^, ^28^ Those with children expressed fear that their children might be taken away if they disclose the abuse.^26^

Some factors that hinder women's disclosure include difficulties in identifying certain forms of abuse, lack of means to leave the abuser, efforts to avoid reliving the trauma, and the belief that abuse was a normal part of being in a relationship.^26^

Similarly, adolescents that are victims of dating violence have to deal with emotional barriers such as embarrassment and shame, minimizing the severity of the situation, fear of retaliations, perceiving help‐seeking as a sign of weakness and the desire to keep the abuse private.^18^

Limited awareness of their rights and unknown of the available services or how healthcare providers could help influence the availability of women and adolescents to confront this issue.^18^, ^25^, ^26^

Individuals with disabilities, survivors who experienced revictimization, multiple or complex traumas with high levels of trauma‐related symptoms, as well as those with cognitive limitations and developmental issues—particularly children—may face greater challenges in engaging with psychosocial interventions.^24^

Specific vulnerabilities of minorities and groups facing structural inequality were studied by three systematic reviews.^23^, ^27^, ^28^ These reviews discussed the specific barriers faced by minority ethnic, and immigrant women who have experienced IPV, showing that their individual experiences of abuse are linked and intensified due to the women's immigration status. In these groups, language barriers, fear of deportation, and controlling and isolating behaviors are frequently imposed by the perpetrators.^23^, ^28^

#### 3.4.2 Facilitators

According to survivors, a family history of violence may encourage help‐seeking behavior and support from family members who have also experienced violence.^18^, ^23^ Among women with children, concerns about their safety and the intention to protect them from future violence influence the decision to seek help.^20^, ^27^ Additionally, the intensification of physical abuse and the deterioration of the relationship with the perpetrator often led to the decision to take urgent action.^27^

The desire to improve their mental health and well‐being influenced the decision to seek healthcare and legal services aimed at supporting their recovery from abuse and enhancing their overall health.^20^ Evidence suggests that resilience and women's efforts to educate themselves about healthy relationships may contribute to the recovery process.^28^

Survivors' familiarity with their rights and their intention to become informed about them contribute to seeking support from these services.^20^ Violence survivors also reported that having control over what and how much they disclosed facilitated the process of disclosure.^26^

Regarding providers personal characteristics one review highlight that gender of the provider influence in the disclosure, in this sense female victims feel more comfortable with females.^26^ In addition, providers feeling informed and adequately prepared to handle IPV cases is described as a contributing factor to addressing this issue, including awareness of IPV dynamic among diverse populations.^19^, ^20^, ^22^, ^26^

One review suggests that some healthcare professionals like obstetricians are more likely to conduct screenings when they have personal experience with IPV or have a close acquaintance who has experienced it^22^ and another one highlight that providers engaging with the community strengthens bonds and supports actions aimed at addressing VAW.^25^

### 3.5 The role of the survivor–healthcare professional relationship in disclosure and addressing VAW

#### 3.5.1 Barriers

**FIGURE 3:**
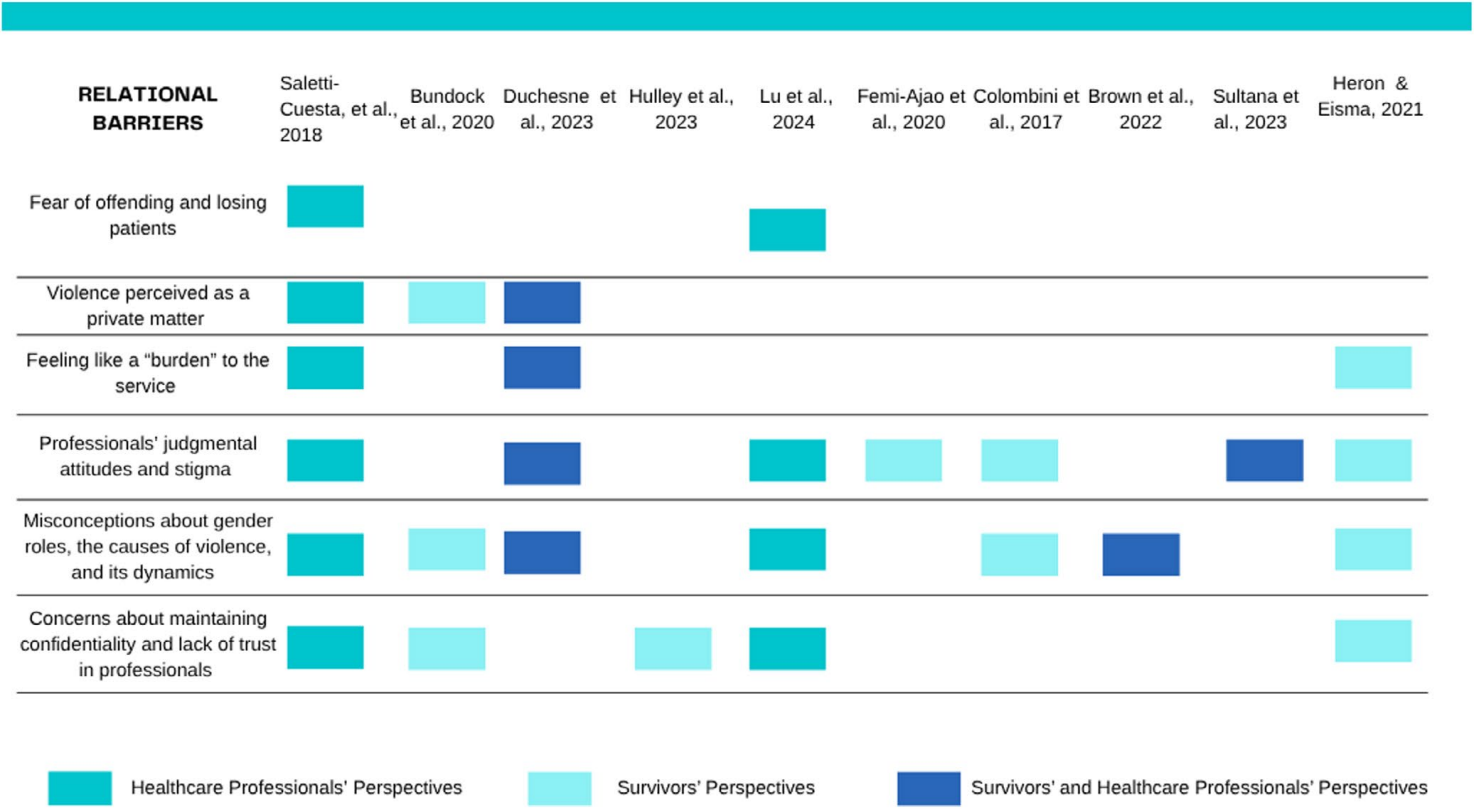
Relational barriers described in the studies.

Figure 3 shows relational barriers from the perspective of providers and VAW survivors. The particularities of interactions between providers and survivors influence the disclosure of violence in healthcare settings. Some studies show that providers' fear of offending patients can limit screening efforts.^22^, ^25^ Providers also expressed concerns such as fear of becoming involved in a personal issue, fear of appearing unprofessional, and consequently losing patients.^25^ Consequently, patients described feeling burdensome and being treated as less important than other patients, particularly ine care settings.^19^

Professional attitudes could limit VAW disclosure among survivors. Providers' perceptions about gender roles, beliefs about violence as a private matter, and their imaginaries in terms of stereotypes moralism, and judgmental attitudes tend to affect their clinical practices.^19^, ^22^, ^25^

Negative attitudes among health workers, such as being judgmental or blaming women who experienced IPV, contributed to making discussions about IPV uncomfortable.^21^ The lack of a positive relationship between patients and providers is a mentioned as a main barrier which attitudes like lack of empathetic behavior, disinterested, avoiding eye contact, or failing to listen were also seen as obstacles.^26^

Among obstetricians, screening was also influenced by personal beliefs about the prevalence of IPV, perceptions of patient risk profiles, and feelings of powerlessness to effect change in patients' lives.^22^

Particularly, adolescents have to deal with the fear that providers might report them to their parents and/or to specialized services such as child protection, which makes it difficult for them to trust adults in professional roles.^18^

Bundock et al.^18^ analyzed studies conducted with adolescents aged 10–24 years. The evidence shows that, overall, this population tends to seek help primarily from informal sources, and when they do turn to formal sources, they are more likely to approach school staff than healthcare providers, social services, or the police. However, the findings also indicate that forms of abuse such as physical and sexual violence are more likely to prompt adolescents to seek support from formal networks than emotional violence. For instance, adolescents tend to access healthcare practitioners in cases of sexual abuse.^18^

Differences related to age and the type of sources accessed according to the type of abuse were observed. For instance, evidence shows that adolescents aged 14–15 years who experienced abuse were more likely to intend to seek help than those aged 16–17 years.^18^ Evidence also shows that adolescents tend to understand violence primarily in relation to physical and sexual abuse.^18^

Among adolescents, particular dynamics of violence related to the intergenerational cycle of violence were observed. In this sense, histories of violence among other female family members influenced their decision to seek help.^18^

Victims' perceptions of the HCP's ability to appropriately respond to a disclosure further influenced their decision to speak out. In addition, survivors tend to be afraid to be judged by the providers, their history not be validated, as well their confidentiality being broken.^26^

#### 3.5.2 Facilitators

A women‐centered care approach that respects women's decision making processes, combined with a biopsychosocial perspective, can guide the provision of more compassionate and supportive care while strengthening the healthcare response.^25^

A comprehensive approach is recognized as essential for understanding survivors from a holistic perspective, contributing to improved access to psychological and social support tailored to their needs.^19^ In this sense, intervention suitability and effectiveness were influenced by how well professionals could adapt their approaches to meet participants' individual needs.^24^

The reviews emphasize that an open relationship with providers based on trust and collaboration, non‐judgmental attitudes, validation of women's experiences, and responsiveness to their safety concerns facilitates interactions between women and healthcare professionals.^19^, ^20^, ^22^, ^24^, ^25^, ^26^

Survivors are more likely to feel comfortable seeking help when providers are validating, informed about the diverse manifestations of IPV, and sensitive to how these experiences may differ across cultural and sexual minority groups.^20^

Emotional support and resource guidance were the primary forms of assistance provided by primary care professionals.^25^ Providing quality information about available resources helps to enhance women's access to their rights and strengthen their autonomy.^19^, ^20^, ^25^

### 3.6 The influence of context: Organizational, structural, and cultural factors and their impact on VAW interventions

#### 3.6.1 Barriers

**FIGURE 4:**
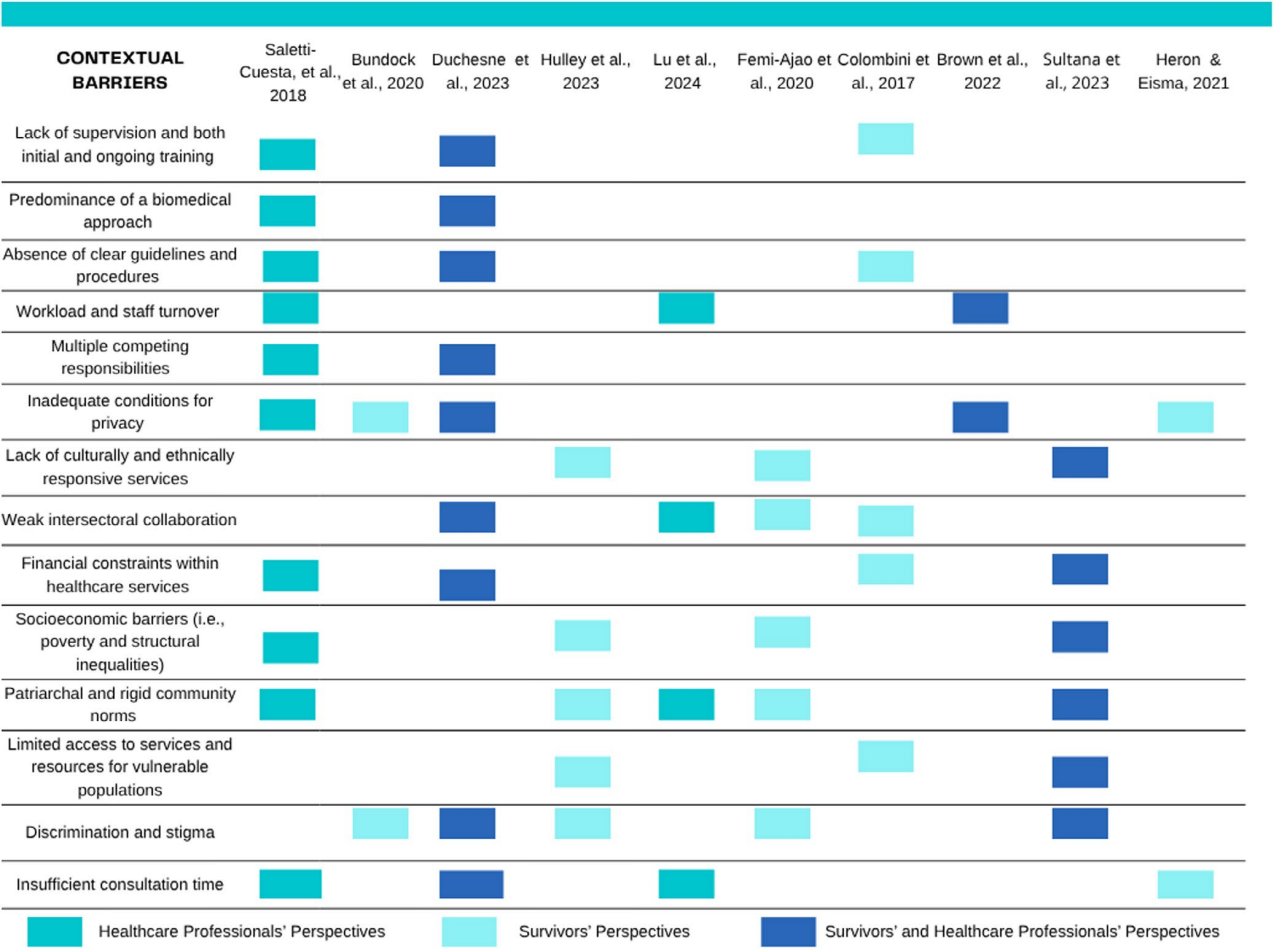
Contextual barriers described in the studies.

Figure 4 shows contextual barriers from the perspective of providers and VAW survivors. The lack of workforce education is one of the main obstacles to addressing this issue in healthcare settings, given the gaps in both initial and ongoing training, and absence of supervision influencing the providers qualified knowledge and understanding of their legal responsibilities in cases of VAW.^21^, ^25^

The biomedical approach is a barrier that tends to limit opportunities to address violence as a health‐related issue. Some studies also identify this paradigm in practices that prioritize physical aspects of care, or that avoid addressing the issue directly by referring women immediately to psychological or social support services.^25^

This approach remains prevalent in healthcare settings, limiting the ability of professionals to address this issue. As a result, limited time is allocated during consultations for screening VAW exposure, compounded by the absence of guidelines or standard procedures, along with heavy workloads, multiple competing responsibilities, and high staff turnover or frequent rotations of staff.^21^, ^24^, ^25^, ^26^

Time constraints impacted the opportunities to address violence in healthcare settings in several of the included studies.^19^, ^22^, ^25^, ^26^ For instance, among obstetricians and gynecologists, screening practices were influenced by the limited time available for consultations and the high number of patients to attend.^22^

On the other hand, time management also emerged as a relevant point from survivors' perspectives. For instance, co‐locating IPV services with other services, such as healthcare, could help mitigate the transportation and time barriers women face in their daily routines.^20^ Other studies reinforce that women perceived healthcare providers as lacking the time needed for them to disclose their full story.^26^

The setting in which the intervention is conducted is a relevant factor, as participants need to feel a sense of physical and emotional security and be protected from further harm. The lack of privacy and concerns about confidentiality also hinder the disclosure of sensitive issues such as violence, particularly among adolescent patients.^18^, ^25^, ^26^

Challenges related to the specialized network and referral systems—including a lack of intersectoral collaboration and limited resources—negatively affect providers' ability to address this issue and ensure proper case follow‐up.^21^, ^25^ Furthermore, integrating the healthcare sector into the specialized network for addressing violence requires consistent financial support and clearly defined roles and responsibilities across sectors, with low‐resource and rural areas being particularly affected.^21^

Poverty and financial crises provoke socio‐economic dependence, limit women's access to essential resources, and contribute to their increased risk of spousal abuse.^23^, ^28^

Minorities and groups that have historically faced structural inequalities, such as immigrants and Black women, encounter several barriers in accessing specialized services and support. Language barriers are frequently reported in the literature as limiting help‐seeking behaviors and access to services, and are sometimes even used by perpetrators to exert further control and power over victims.^23^, ^27^, ^28^

In addition, these groups face cultural barriers related to rigid norms rooted in patriarchal gender ideologies, community influences, family honor and influences of religion, which affect their ability to recognize the violence and influence their decisions to leave abusive relationships.^23^, ^27^, ^28^

Immigration issues are also reported as barriers including fears of deportation, lack of familiarity with the legal system and their rights, fear of losing custody of their children, difficulties with documentation and interacting with legal institutions.^23^, ^27^, ^28^

Experiences of discrimination, stereotypes, and structural racism were also described in the studies, contributing to high levels of isolation and limiting access to specialized services, particularly among Black women.^23^, ^27^, ^28^ In addition, the lack of culturally and ethnically responsive services, combined with insufficient information and limited proactive engagement by support systems, presents significant obstacles that affect the use of these services.^23^

#### 3.6.2 Facilitators

Organizational factors associated with healthcare workforce education are described as factors that enable the addressing of VAW interventions in healthcare settings.^21^, ^25^

The capacity of services to provide a confidential and safe space can be ensured by enhancing physical infrastructure, streamlining client flow, and updating policies.^21^, ^26^ Among adolescents and young people, services that provide a friendly, safe, and confidential environment with empathetic staff are highly valued and play an important role in their willingness to use these facilities.^18^, ^24^

Teamwork, intersectoral collaboration that integrates local‐ and national‐level networks, strong coordination across the health sector, and the incorporation of IPV support into multi‐service environments — including organized referral pathways and effective service networks — have been identified as key facilitators that promote survivors' disclosure and enhance the effectiveness of interventions.^20^, ^21^, ^25^

Guidelines and protocols, along with managerial leadership and the presence of an “IPV champion,” have been recognized as critical enablers of a more comprehensive response.^21^

Technological support is described by women survivors as essential for improving access to prevention programs, access to services and education, thereby expanding opportunities for support and information.^20^, ^28^

One review recognized that providing services in multiple languages, the availability of resources within healthcare settings supported survivors' access to assistance facilitated help‐seeking.^20^ Situating the services in easily accessible locations,^20^, ^24^ informational materials, such as leaflets and posters on domestic violence, in waiting rooms contributed to facilitating victims' disclosure^26^ are among the strategies identified as contributing to victims' disclosure.

The format of the intervention is also highlighted, with positive relationships formed in group settings identified as important enablers of recovery.^24^ Similarly, outside the intervention setting, several studies have noted that informal support from family, friends, and the community can facilitate survivors' formal help‐seeking and improve access to specialized services.^19^, ^20^, ^24^

Institutional support is essential for the effectiveness of the interventions. This support includes financing and budget allocation,^21^ time to screen and to provide an appropriate response,^19^, ^22^, ^25^, ^26^ and the creation of a support network for providers preventing work‐related condition (e.g. vicarious trauma and burnout).^19^

A women‐centered approach is highlighted as a relevant framework that reinforces the importance of providers and women's relationships, as well as considers women's autonomy, expectations, and needs as priorities of the interventions.^19^, ^20^, ^25^

Overall, the studies described that first‐line support actions should focus on empathetic listening, validating women's experiences and feelings, ensuring safety, and providing information and referrals within their communities. Evidence also shows that community engagement and multisectoral collaboration, grounded in clarity regarding the roles and responsibilities of each sector, improve surveillance and help ensure that the most appropriate responses are provided.^21^

## 4 DISCUSSION

This review examines the barriers and facilitators related to implementing interventions that address VAW within health services. To effectively inform policies and decision‐makers, it is crucial to provide a comprehensive overview of the available evidence on the key factors that enable or hinder the implementation of such interventions.

Similar to other reviews of interventions in low‐ and middle‐income countries, common system‐level obstacles generally include a lack of knowledge and training, overburdened and under‐resourced systems, prevailing patriarchal norms, the social acceptance of gender‐based violence, and society's limited preparedness to address the issue.^9^, ^29^

VAW and adolescents remain a complex issue involving diverse challenges. In healthcare settings, the biomedical model of care remains the dominant framework, often limiting healthcare professionals' practices and the development of competencies to address such issues.^3^

Even when healthcare professionals recognize violence as a relevant health issue, structural and system level factors still influence how the problem is addressed.^3^ Health systems face several challenges, including insufficient healthcare personnel, limited funding and privacy, and a lack of leadership support.^29^

Our findings highlight that providers face challenges related to role ambiguity, stemming from unclear definitions of their professional scope.^30^ Evidence suggests that healthcare professionals report difficulties in addressing this issue due to insufficient technical training, fear of breaching patient confidentiality, and the perception that this topic does not fall within their responsibilities or professional competence.^3^

Providers reported difficulties navigating the intersection between their personal and professional identities in this context, particularly when strong emotions resurfaced, making it challenging to maintain a professional stance. Additionally, providers sometimes held stereotypical beliefs about patients affected by IPV, which led to discrimination and stigma.^19^

At the same time, the role conflict experienced by providers is influenced by various structural factors. Limited time to address all patients' needs, inadequate resources, and personal discomfort may compromise the effectiveness of interventions.^29^, ^31^ A lack of time and privacy, as well as insufficient guidelines and standardized procedures, are commonly reported issues that hinder screening practices, according to providers' perceptions.^3^

Studies analyzed tend to identify several challenges that healthcare providers face when dealing with violence, which also affects their own well‐being, particularly due to the emotional impact that VAW evokes in them. Regarding these emotional impacts, some coping strategies have been described, such as mentoring, leisure‐time activities, and strengthening bonds with communities.^25^

However, it is important to highlight that individual coping strategies must be accompanied by institutional policies, such as workforce education and continuous training, emotional support programs for providers, and supervision.^21^, ^25^ In this sense, it is essential for providers to recognize that they are not alone in confronting such a complex issue.

As part of the essential training of healthcare professionals, competencies for managing violence across the life course should be incorporated into the medical curriculum. For instance, a study conducted among obstetrics and gynecology residents shows that addressing these gaps in education and training is essential, given the persistently low screening rates observed in healthcare settings despite the high prevalence of domestic violence among antenatal care attendees.^32^

On the other hand, considering the cycle of IPV and common patterns of aggressor behavior, concerns about safety are frequently reported by both women and healthcare providers. Re‐victimization and vicarious violence against children, family members, and friends discourage women from disclosing the abuse, while fear of possible retaliation discourages providers from conducting screenings and, consequently, from offering further interventions.^3^

Hence, several obstacles were also highlighted across the reviews, including the personal experiences, feelings, and beliefs of both providers and survivors, as well as the impact of their interactions.

The relationship between providers and women revealed that self‐confidence and trust were key concepts frequently discussed across the included studies. These concepts emerged in two main ways. First, healthcare providers often reported a lack of confidence stemming from insufficient competence to address the issue. Second, women perceived that providers might not be able to handle their disclosure of violence appropriately or respond adequately to their needs, which negatively impacted their help‐seeking behaviors.

It is essential that healthcare managers support structural changes to confront the barriers present in healthcare settings. In this sense, evidence shows the urgency of shifting paradigms toward a biopsychosocial approach, women‐centred care, empowerment support, and respect for women's autonomy.^25^ For instance, time management is a relevant aspect to assess in this context. Restricted consultation time shapes care delivery, leading providers to prioritize other clinical demands and consequently relegate this issue to a minor concern within the healthcare context. This limitation was also perceived by survivors as an obstacle to disclosing their experiences and, in turn, to building an empathic relationship with providers.

The inclusion of resources within healthcare services is also necessary, as it is more likely that in some cases survivors will seek healthcare services rather than specialized violence services.^20^ Some strategies to facilitate disclosure describe how providers can identify signs of violence by using various opportunities during encounters with women, such as routine visits and health promotion activities.^25^

In line with our findings a review of interventions for pregnant women in low‐ and middle‐income countries highlights various key factors that facilitate implementation of interventions. In this sense, providers' attitudes (e.g., being non‐judgmental, empathetic, and supportive), the development of strong relationships based on trust, honesty, and openness between women and service providers, and organizational commitment all contribute to the success of such initiatives.^9^

Regarding women's personal factors, and in line with our findings, evidence suggests that a lack of autonomy, economic hardship, financial dependence on husbands, cultural representations of family, and fear of retaliation that impedes taking legal action are key factors that prevent women from addressing this issue.^9^, ^29^, ^33^, ^34^

Moreover, cultural representations of family and intimate partner relationships, along with difficulties in recognizing forms of abuse, affect survivors' help‐seeking behavior.^9^ Evidence shows that, beyond the barriers commonly faced by survivors of violence, migrant women encounter additional challenges in accessing healthcare services, such as lack of information, language barriers, economic status, immigration‐related issues, discrimination and cultural differences.^35^

Social imaginaries about what is understood as violence influence adolescents' attitudes toward confronting violent experiences. In this regard, it is important to develop campaigns and educational programs that contribute to changing adolescents' representations of violence, helping them identify signs of abuse and prevent both exposure to and perpetration of violence.

Considering that adolescents were likely to access informal sources to deal with experiences of violence, it is necessary for services to develop comprehensive, non‐judgmental, and empathetic approaches to support adolescents exposed to violence. An intersectoral approach is necessary, given the need for communication and interprofessional collaboration among school staff, social services, and healthcare providers.

In addition, considering the obstacles disclosed by adolescents, recommendations include being clear about confidentiality and its limits, explaining what will happen with their testimony, and providing strategies to ensure safety before disclosure.^18^

Overall, women and girls tend to face diverse obstacles in accessing quality healthcare services. An observational study conducted in Latin American countries among women aged 15–49 years showed the presence of geographical and financial barriers, as well as constraints such as not wanting to attend healthcare services alone and not being permitted to go alone.^36^

These findings also highlight various forms of structural violence shaped by gender norms, stigma, fear of reprisals, and socioeconomic vulnerabilities, which limit women's opportunities to access healthcare services and, consequently, to disclose experiences of violence within domestic and intimate partner relationships.

The opportunity to build strong relationships between healthcare providers and patients, particularly in settings frequented by vulnerable groups (e.g., adolescents, migrants, and Black women), creates valuable conditions for addressing sensitive issues.^37^ In this sense, it is crucial that healthcare services—such as primary health care, emergency department, maternal services and community‐based services—strengthen their capacity to reach these populations.^38^ Facilitating access to and strengthening the capacities of primary health care is important, as visits to these facilities are less likely to raise suspicion from aggressors.

In addition, evidence from low‐ and middle‐income countries such as Malaysia, South Africa, and India has shown that “IPV champions” can be an effective strategy within healthcare services, including primary healthcare settings and emergency departments, to motivate and support other professionals.^21^

The introduction of champions in the context of domestic violence is an emerging topic; however, their role could contribute significantly by mentoring and supporting healthcare professionals in addressing gender‐based violence in their practice, as well as by providing emotional support for staff.^39^

In these scenarios, confronting VAW requires comprehensive, multisectoral responses, with healthcare professionals playing a vital role in addressing psychosocial issues.^4^ Although this is a phenomenon that demands intersectoral approaches, a recurring challenge is the lack of coordination among services where victims seek assistance, which hinders the provision of comprehensive care for survivors and their dependents.^3^

Globally diverse models and guides are being produced to be implemented in specific settings like family planning and other reproductive and sexual health care settings to confront diverse forms of gender‐based violence including sexual violence and reproductive coercion.

Changes in healthcare service delivery models that improve how gender‐based violence is addressed remain necessary. For instance, experiences from low‐ and middle‐income countries highlight the implementation of one‐stop centers to respond to intimate partner violence and sexual violence.

Sikder et al.^40^ document the use of this model of care in LMICs (Bangladesh, Nepal, Sri Lanka, and Rwanda), where comprehensive case management for survivors is provided by integrating health, welfare, counseling, and legal services in a single location, most often within hospitals. In addition, a recent systematic review of this model shows enablers such as standardized policies and procedures, along with regular interagency meetings that promote more effective multisectoral coordination.^41^

However, greater demands in terms of infrastructure, financial resources, and the limited capacity to address other forms of violence and populations beyond cisgender women may pose obstacles to implementation when compared with other integrated models.^41^ More robust evidence is still required to identify the potential of this model across diverse settings and populations, particularly those exposed to socioeconomic vulnerabilities and historical inequalities.

In addition, a review that explored the experiences of women seeking care at first‐contact healthcare facilities in South Africa after sexual violence and during follow‐up care also contributes to this discussion.^42^ The findings highlight women's experiences within healthcare facilities, particularly in Thuthuzela Care Centres, which provide legal support, social assistance, medical care, and mental health assessments for survivors within a victim‐centred model of care.^42^

In this context, underreporting is identified as one of the key obstacles, limiting access to appropriate care (e.g., pregnancy‐related counseling, information on sexually transmitted infections, and post‐exposure prophylaxis). Randa et al.^42^ emphasize that, beyond existing legislation and protocols, a holistic and integrated approach is necessary, taking into account evidence on survivors' pathways both within and outside these specialized centers.

The Health Systems Wheel framework proposed by the WHO outlines components that must be strengthened to ensure equitable and efficient service delivery. These components include: (i) leadership and governance, (ii) multisectoral coordination, (iii) workforce development, (iv) healthcare delivery, (v) infrastructure, (vi) financing, and (vii) monitoring and evaluation.^17^

Based on these components, some recommendations are summarized, with a focus on strengthening system capacities and addressing violence through the incorporation of facilitators and lessons learned that support the implementation of evidence‐based interventions. More details on this study's implications for practice, policy, and research are shown in Table 2.

**TABLE 2:** Implications for practice, research, and policies.

Level	Recommendation
Practice	To establish effective rapport and use validated tools to address sensitive topics. Ensuring confidentiality, comfort, and safety can facilitate disclosure^24^, ^26^, ^28^ Early prevention of violence, providing emotional support and information, assessing risk and planning for safety, and coordinating referrals within the team or with external services are key components of care^21^, ^25^ Providers' attitudes—such as empathy, validation, non‐judgmental approaches, and non‐pressuring behavior—should be actively promoted^24^, ^26^ To ensure clear, culturally appropriate, and accessible language^18^ Respecting women's autonomy through high‐quality, informed care based on individual needs is essential Providing information on women's rights, available resources for survivors (e.g., shelters, legal advocacy, counseling), and facilitating access to mental health support^21^, ^26^
Research	Studies must contribute to deeper understanding of how the gendered social roots of VAW, organizational and contextual factors influence and maintaining the barriers^25^ Produce evidence based on robust methodological design (e.g., longitudinal, clinical trials, mixed‐methods and cross‐cultural studies) to better understand help‐seeking behaviors and to guide policy development from the perspectives of survivors, their supporters, and practitioners^18^, ^22^, ^23^ To create and validate a facilitator of help‐seeking scale based on robust studies to assess potential factors that mediate or moderate help‐seeking behavior^21^ Expand the studies to forms of abuse less explored and subtle (e.g. emotional and coercive control) and their dynamics^18^ Develop research about help‐seeking behavior and critical pathways among vulnerable groups and diverse populations such as adolescents, LGBTQIA+ youth, individuals with disabilities^18^, ^22^ as well as those groups that experienced health inequities such as immigrants^21^, ^27^ and Black women^27^ The inclusion of analysis based on gender, sexual orientation, race, ethnicity, and disability is a relevant topic to be address on the researches^19^ Detailed reporting of implementation strategies in future intervention studies is critical to inform scale‐up efforts^23^ Identify lesson learned through the interventions, aimed to apply the facilitators according to every context^23^ Future research must prioritize interventions conducted in low‐ and middle‐income countries To fully assess the intervention's relevance and reach, it is essential to consider the perspectives of individuals who do not engage with or complete it^22^ Encourage further research into diverse and comprehensive topics to design culturally sensitive interventions that support help‐seeking behavior among immigrant women considering the impact of sociocultural factor in the implementation of the interventions^28^
Policies	Policies must ensure adequate financial resources to address violence in healthcare settings,^19^ as well as broader policies that respond to women's needs (e.g., employment opportunities, legal assistance, housing, and financial support)^20^ The implementation of care models grounded in reflective, woman‐centered, gender‐sensitive, and rights‐based approaches is essential when addressing sensitive topics The promotion of culturally appropriate interventions is strongly recommended. These should be culturally responsive, anti‐racist, multilingual, and offer long‐term support for women^20^, ^21^, ^28^ Developing a comprehensive approach within healthcare systems and ensuring ongoing organizational support for providers is critical^23^ Workforce education and continuous training should focus on patterns and complexities of violence, particularly among diverse and vulnerable populations. This includes how to initiate conversations about IPV, respond to disclosures, understand legal responsibilities and outcomes, recognize internalized bias, and provide trauma‐informed care^19^, ^21^, ^24^, ^26^, ^27^ Campaigns and engagement from the healthcare sector are needed to raise public awareness and address misconceptions about abuse, encourage help‐seeking, inform citizens of their rights, and promote knowledge of available services. These campaigns should also address emerging forms of abuse, such as digital violence, especially among young populations^18^ Youth‐friendly and safe services for adolescents are essential^18^ Initiatives that support healthcare providers' well‐being should be developed, particularly those that help them cope with emotional challenges when addressing sensitive topics or confronting their own experiences with violence. Burnout prevention and vicarious trauma mitigation are highly recommended^19^ The creation of safe environments, ensuring privacy, adequate staffing, and service adaptations to meet survivors' needs is essential^19^, ^21^ Collaboration between survivors and experts in the development of educational programs is key to ensuring services are survivor‐informed and policies better aligned with their needs^19^ Intersectoral collaboration and effective referral systems—both within and beyond healthcare—are necessary to address physical and mental health needs as well as legal involvement^19^

### 4.1 Limitations and strengths

The main limitation concerns the context in which the evidence was produced. Most of the included reviews are based on primary studies conducted in high‐income countries. This may limit the generalizability of the findings and the applicability of the recommendations, given the financial and human resource constraints commonly faced by low‐ and middle‐income countries when implementing such interventions. Therefore, the recommendations should be applied with caution and adapted to the specific context. Another limitation is the exclusion of gray literature from the search process. Nonetheless, a comprehensive search across databases was conducted to ensure a broad and exhaustive inclusion of peer‐reviewed publications.

The present study focuses exclusively on the female population, recognizing that violence against girls and women remains a persistent global concern. Young women and adolescents, in particular, are especially vulnerable to various forms of violence, which can lead to adverse outcomes for their sexual and reproductive health. Although this choice aligns with the study's objectives and reflects the worldwide prevalence of violence against women and girls and its detrimental effects on health, it excludes men and boys from the analysis.

The research team's decision to exclude the male population is based on the understanding that including these groups would require specific analytical frameworks to adequately examine how violence affects them, including considerations related to the intergenerational cycle of violence, the specific dynamics of perpetrator–victim roles, social imaginaries surrounding violence, and the obstacles men face in accessing healthcare services. In this sense, we acknowledge the importance of generating evidence on masculinities and violence in diverse contexts, as well as understanding the barriers to addressing these issues within healthcare settings.

As strengths, this review provides a comprehensive overview of the key factors that enable or hinder the implementation of interventions aimed at addressing VAW in healthcare settings. The use of evidence synthesis, based on systematic and rigorous methods of search and data analysis, contributes to the development of evidence‐based recommendations that may enhance the benefits and positive impacts of such interventions. Moreover, critically analyzing the factors that undermine effectiveness can help improve interventions by incorporating the perspectives of both providers and survivors.

## 5 CONCLUSIONS

The synthesis of the evidence highlights that the implementation of interventions requires a comprehensive approach that takes into account the complex nuances influencing the success of efforts to address VAW in healthcare settings. The intersection of individual, relational, organizational, and sociocultural factors significantly impact the effectiveness of these interventions. Therefore, efforts to strengthen initiatives in this sector must take all of these factors—and their complexities—into account.

## AUTHOR CONTRIBUTIONS

Design and planning: ORS, LR, EZ and FGS. Data selection and extraction: ORS, LR and EZ. Analysis, interpretation, writing the paper and critical review of its content: ORS, LR, EZ, and FGS. Supervision: FGS. All the authors approved the final version.

## FUNDING INFORMATION

This study was financed in part by the National Council for Scientific and Technological Development—CNPq and the Department of Science and Technology of Secretariat of Science, Technology, Innovation and Health Complex of Ministry of Health of Brazil—MoH. Project number 444414/2023–1 CNPq. Proposal Call No. 21/2023—Transdisciplinary Studies in Public Health.

## CONFLICT OF INTEREST STATEMENT

The authors declare that they have no competing interests with this work.

## Supporting information


**Table S1.** Search strategy.
**Table S2.** JBI Critical Appraisal Checklist for systematic reviews and research syntheses.

## Data Availability

The datasets used and/or analyzed during the current study are available from the corresponding author on reasonable request.

## References

[1] United Nations High Commissioner for Human Rights . Declaration on the elimination of violence against women: proclaimed by General Assembly resolution 48/104 of 20 December 1993. Accessed June 8, 2025. https://digitallibrary.un.org/record/179739?v=pdf

[2] World Health Organization . Violence against Women Prevalence Estimates, 2018: Global, Regional and National Prevalence Estimates for Intimate Partner Violence against Women and Global and Regional Prevalence Estimates for Non‐partner Sexual Violence against Women. World Health Organization; 2021.

[3] D'oliveira AFPL , Pereira S , Bacchus LJ , et al. Are we asking too much of the health sector? Exploring the readiness of Brazilian primary healthcare to respond to domestic violence against women. Int J Health Policy Manag. 2022;11(7):961‐972. 33327691 10.34172/ijhpm.2020.237 PMC9808197

[4] García‐Moreno C , Hegarty K , d'Oliveira AF , Koziol‐McLain J , Colombini M , Feder G . The health‐systems response to violence against women. Lancet. 2015;385(9977):1567‐1579. 25467583 10.1016/S0140-6736(14)61837-7

[5] Hoelle RM , Elie MC , Weeks E , et al. Evaluation of healthcare use trends of high‐risk female intimate partner violence victims. West J Emerg Med. 2015;16(1):107‐113. 25671018 10.5811/westjem.2014.12.22866 PMC4307692

[6] Bonomi AE , Anderson ML , Rivara FP , Thompson RS . Health care utilization and costs associated with physical and nonphysical‐only intimate partner violence. Health Serv Res. 2009;44:1052‐1067. 19674432 10.1111/j.1475-6773.2009.00955.x PMC2699921

[7] García‐ Moreno C , Jansen HA , Ellsberg M , Heise L , Watts C . WHO Multi Country Study on women's Health and Domestic Violence against Women: Initial Results on Prevalence, Health Outcomes and women's Responses. Summary report. World Health Organization; 2005.

[8] Jahanfar S , Howard LM , Medley N . Interventions for preventing or reducing domestic violence against pregnant women. Cochrane Database Syst Rev. 2014;2014(11):CD009414. 25390767 10.1002/14651858.CD009414.pub3 PMC7104547

[9] Sapkota D , Baird K , Saito A , Anderson D . Interventions for reducing and/or controlling domestic violence among pregnant women in low‐ and middle‐income countries: a systematic review. Syst Rev. 2019;8(1):79. 30940204 10.1186/s13643-019-0998-4 PMC6889323

[10] Prosman GJ , Lo Fo Wong SH , Van Der Wouden JC , Lagro‐Janssen ALM . Effectiveness of home visiting in reducing partner violence for families experiencing abuse: a systematic review. Fam Pract. 2015;32(3):247‐256. 25947931 10.1093/fampra/cmu091

[11] Van Parys AS , Verhamme A , Temmerman M , Verstraelen H . Intimate partner violence and pregnancy: a systematic review of interventions. PLoS One. 2014;9(1):e85084. 24482679 10.1371/journal.pone.0085084 PMC3901658

[12] El Morr C , Layal M . Effectiveness of ICT‐based intimate partner violence interventions: a systematic review. BMC Public Health. 2020;20(1):1372. 32894115 10.1186/s12889-020-09408-8 PMC7476255

[13] Moore GF , Audrey S , Barker M , et al. Process evaluation of complex interventions: Medical Research Council guidance. BMJ. 2015;350:h1258. 25791983 10.1136/bmj.h1258 PMC4366184

[14] Aromataris E , Fernandez R , Godfrey C , Holly C , Khalil H , Tungpunkom P . Umbrella Reviews (2020). In: Aromataris E , Lockwood C , Porritt K , Pilla B , Jordan Z , eds. JBI Manual for Evidence Synthesis. JBI; 2024. doi:10.46658/JBIMES-24-08

[15] Langlois ÉV , Daniels K , Akl EA , eds. Evidence Synthesis for Health Policy and Systems: A Methods Guide. World Health Organization; 2018. 33877749

[16] Brazil. Ministry of Health. Secretariat of science, technology, innovation, and strategic health supplies. Department of Science and Technology . Methodological Guideline: Evidence Synthesis for Policies. Ministry of Health; 2020.

[17] World Health Organization . Monitoring the Building Blocks of Health Systems. WHO Document Production Services; 2010.

[18] Bundock K , Chan C , Hewitt O . Adolescents' help‐seeking behavior and intentions following adolescent dating violence: a systematic review. Trauma Violence Abuse. 2020;21(2):350‐366. 29683049 10.1177/1524838018770412

[19] Duchesne E , Nathoo A , Walker M , Bartels SA . Patient and provider emergency care experiences related to intimate partner violence: a systematic review of the existing evidence. Trauma Violence Abuse. 2023;24(5):2901‐2921. 35997064 10.1177/15248380221118962 PMC10594849

[20] Ravi KE , Robinson SR , Schrag RV . Facilitators of formal help‐seeking for adult survivors of IPV in the United States: a systematic review. Trauma Violence Abuse. 2022;23(5):1420‐1436. 33685292 10.1177/1524838021995954

[21] Colombini M , Dockerty C , Mayhew SH . Barriers and facilitators to integrating health service responses to intimate partner violence in low‐ and middle‐income countries: a comparative health systems and service analysis. Stud Fam Plan. 2017;48(2):179‐200. 10.1111/sifp.12021 PMC5518204 28422291

[22] Lu C , Georgousopoulou E , Baloch S , et al. Identifying the barriers faced by obstetricians and registrars in screening or enquiry of intimate partner violence in pregnancy: a systematic review of the primary evidence. Aust N Z J Obstet Gynaecol. 2024;64(1):19‐27. 37786258 10.1111/ajo.13747

[23] Hulley J , Bailey L , Kirkman G , et al. Intimate partner violence and barriers to help‐seeking among black, Asian, minority ethnic and immigrant women: a qualitative Metasynthesis of global research. Trauma Violence Abuse. 2023;24(2):1001‐1015. 35107333 10.1177/15248380211050590 PMC10012394

[24] Brown SJ , Carter GJ , Halliwell G , et al. Survivor, family and professional experiences of psychosocial interventions for sexual abuse and violence: a qualitative evidence synthesis. Cochrane Database Syst Rev. 2022;10(10):CD013648. 36194890 10.1002/14651858.CD013648.pub2 PMC9531960

[25] Saletti‐Cuesta L , Aizenberg L , Ricci‐Cabello I . Opinions and experiences of primary healthcare providers regarding violence against women: a systematic review of qualitative studies. J Fam Violence. 2018;33(6):405‐420.

[26] Heron RL , Eisma MC . Barriers and facilitators of disclosing domestic violence to the healthcare service: a systematic review of qualitative research. Health Soc Care Community. 2021;29(3):612‐630. 33440034 10.1111/hsc.13282 PMC8248429

[27] Femi‐Ajao O , Kendal S , Lovell K . A qualitative systematic review of published work on disclosure and help‐seeking for domestic violence and abuse among women from ethnic minority populations in the UK. Ethn Health. 2020;25(5):732‐746. 29514473 10.1080/13557858.2018.1447652

[28] Sultana R , Ozen‐Dursun B , Femi‐Ajao O , Husain N , Varese F , Taylor P . A systematic review and meta‐synthesis of barriers and facilitators of help‐seeking behaviors in south Asian women living in high‐income countries who have experienced domestic violence: perception of domestic violence survivors and service providers. Trauma Violence Abuse. 2023;24(5):3187‐3204. 36250293 10.1177/15248380221126189 PMC10594840

[29] Lewis NV , Munas M , Colombini M , et al. Interventions in sexual and reproductive health services addressing violence against women in low‐income and middle‐income countries: a mixed‐methods systematic review. BMJ Open. 2022;12(2):e051924. 10.1136/bmjopen-2021-051924 PMC8867339 35193906

[30] d'Oliveira AFPL , Pereira S , Schraiber LB , et al. Obstacles and facilitators to primary health care offered to women experiencing domestic violence: a systematic review. Interface. 2020;24:e190164.

[31] Sabloak T , Ryan I , Nahi S , Eucalitto P , Simon MA , Premkumar A . Intimate partner violence detected during abortion‐related visits: a systematic review of screenings and interventions. Am J Perinatol. 2024;41(12):1697‐1705. 38365213 10.1055/s-0044-1779746

[32] Labre DM , Del Risco Sánchez O , Monteiro I , Freitas‐Jesus JV , Surita FG . Addressing domestic violence by obstetric and gynecological resident doctors: pre‐post intervention study. BMC Med Educ. 2025;25(1):1405. 41077576 10.1186/s12909-025-08010-z PMC12516890

[33] Daley D , McCauley M , Van Den Broek N . Interventions for women who report domestic violence during and after pregnancy in low‐ and middle‐income countries: a systematic literature review. BMC Pregnancy Childbirth. 2020;20(1):141. 32138721 10.1186/s12884-020-2819-0 PMC7059681

[34] Sánchez OR , Vale DB , Rodrigues L , Surita FG . Violence against women during the COVID‐19 pandemic: an integrative review. Int J Gynaecol Obstet. 2020;151(2):180‐187. 32880941 10.1002/ijgo.13365 PMC9087782

[35] Pérez‐Sánchez M , Immordino P , Romano G , Giordano A , García‐Gil C , Morales F . Access of migrant women to sexual and reproductive health services: a systematic review. Midwifery. 2024;139:104167. 39243595 10.1016/j.midw.2024.104167

[36] Houghton N , Báscolo E , Jara L , et al. Barriers to access to health services for women and children in Latin America. Rev Panam Salud Publica. 2022;46:e94. 35875315 10.26633/RPSP.2022.94 PMC9299390

[37] Surita FG , Sánchez ODR . Routine enquiry for domestic violence during antenatal care: an opportunity to improve Women's health. Rev Bras Ginecol Obstet. 2022;44(3):211‐213. 35576935 10.1055/s-0042-1742735 PMC9948041

[38] Feder GS , Hutson M , Ramsay J , Taket AR . Women exposed to intimate partner violence: expectations and experiences when they encounter health care professionals: a meta‐analysis of qualitative studies. Arch Intern Med. 2006;166(1):22‐37. 16401807 10.1001/archinte.166.1.22

[39] Saberi E , Hurley J , Hutchinson M . The role of champions in leading domestic violence and abuse practice improvement in health care: a scoping review. J Nurs Manag. 2022;30(6):1658‐1666. 34798682 10.1111/jonm.13514

[40] Sikder SS , Ghoshal R , Bhate‐Deosthali P , Jaishwal C , Roy N . Mapping the health systems response to violence against women: key learnings from five LMIC settings (2015–2020). BMC Womens Health. 2021;21(1):360. 34629077 10.1186/s12905-021-01499-8 PMC8504083

[41] Olson RM , García‐Moreno C , Colombini M . The implementation and effectiveness of the one stop centre model for intimate partner and sexual violence in low‐ and middle‐income countries: a systematic review of barriers and enablers. BMJ Glob Health. 2020;5(3):e001883. 10.1136/bmjgh-2019-001883 PMC7170420 32337076

[42] Randa MB , McGarry J , Griffiths S , Hinsliff‐Smith K . Accessing care services after sexual violence: a systematic review exploring experiences of women in South Africa. Curationis. 2023;46(1):e1‐e10. 10.4102/curationis.v46i1.2405 PMC10623477 37916664

