# Supplementary material for: Interventions addressing violence against women in health services: An overview of systematic reviews regarding barriers and facilitators to implementation
Source: Int J Gynaecol Obstet. 2026 Jan 5;174(1):17–33. doi: 10.1002/ijgo.70796 (PMC13278641; doi:10.1002/ijgo.70796)
Supplement: Supplementary file 1 — Table S1. Search strategy. Table S2. JBI Critical Appraisal Checklist for systematic reviews and research syntheses. [file IJGO-174-17-s001.docx]

**Table S1.** Search Strategy

| **Search** | **Actions** | **Details** | **Query** | **Results** | **Time** |
| --- | --- | --- | --- | --- | --- |
| #12 |  |  | Search: **(((((Women[MeSH Terms]) OR (Women[Title/Abstract] OR Girls[Title/Abstract] OR Girl[Title/Abstract] OR Woman[Title/Abstract] OR "Women Groups"[Title/Abstract])) OR (Female[MeSH Terms])) OR (Female[Title/Abstract] OR Females[Title/Abstract])) AND (((((Domestic Violence[MeSH Terms]) OR ("Domestic Violence"[Title/Abstract] OR "Violence, Domestic"[Title/Abstract] OR "Family Violence"[Title/Abstract] OR "Violence, Family"[Title/Abstract])) OR ("Violence Against Women"[Title/Abstract])) OR ((Gender-Based Violence[MeSH Terms]) OR ("Gender-Based Violence"[Title/Abstract] OR "Gender Based Violence"[Title/Abstract] OR "Violence, Gender-Based"[Title/Abstract] OR "Dowry Death"[Title/Abstract] OR "Dowry Deaths"[Title/Abstract]))) OR ((Intimate Partner Violence[MeSH Terms]) OR ("Intimate Partner Violence"[Title/Abstract] OR "Partner Violence, Intimate"[Title/Abstract] OR "Violence, Intimate Partner"[Title/Abstract] OR "Intimate Partner Abuse"[Title/Abstract] OR "Abuse, Intimate Partner"[Title/Abstract] OR "Dating Violence"[Title/Abstract] OR "Violence, Dating"[Title/Abstract])))) AND ((("barriers and facilitators"[Title/Abstract]) OR ("barrier and facilitator"[Title/Abstract])) OR ((barriers[Title/Abstract] OR barrier[Title/Abstract]) AND (facilitators[Title/Abstract] OR facilitator[Title/Abstract])))**  ("Women"[MeSH Terms] OR ("Women"[Title/Abstract] OR "Girls"[Title/Abstract] OR "Girl"[Title/Abstract] OR "Woman"[Title/Abstract] OR "Women Groups"[Title/Abstract]) OR "Female"[MeSH Terms] OR ("Female"[Title/Abstract] OR "Females"[Title/Abstract])) AND ("Domestic Violence"[MeSH Terms] OR ("Domestic Violence"[Title/Abstract] OR "violence domestic"[Title/Abstract] OR "Family Violence"[Title/Abstract] OR "violence family"[Title/Abstract]) OR "Violence Against Women"[Title/Abstract] OR ("gender based violence"[MeSH Terms] OR ("gender based violence"[Title/Abstract] OR "gender based violence"[Title/Abstract] OR "violence gender based"[Title/Abstract] OR "Dowry Death"[Title/Abstract] OR "Dowry Deaths"[Title/Abstract])) OR ("Intimate Partner Violence"[MeSH Terms] OR ("Intimate Partner Violence"[Title/Abstract] OR "partner violence intimate"[Title/Abstract] OR "violence intimate partner"[Title/Abstract] OR "Intimate Partner Abuse"[Title/Abstract] OR "abuse intimate partner"[Title/Abstract] OR "Dating Violence"[Title/Abstract] OR "violence dating"[Title/Abstract]))) AND ("barriers and facilitators"[Title/Abstract] OR "barrier and facilitator"[Title/Abstract] OR (("barriers"[Title/Abstract] OR "barrier"[Title/Abstract]) AND ("facilitators"[Title/Abstract] OR "facilitator"[Title/Abstract])))  **Translations**  **Women[MeSH Terms]:** "women"[MeSH Terms]  **Female[MeSH Terms]:** "female"[MeSH Terms]  **Domestic Violence[MeSH Terms]:** "domestic violence"[MeSH Terms]  **Gender-Based Violence[MeSH Terms]:** "gender-based violence"[MeSH Terms]  **Intimate Partner Violence[MeSH Terms]:** "intimate partner violence"[MeSH Terms] | [198](https://pubmed.ncbi.nlm.nih.gov/?term=%28%28%28%28%28Women%5BMeSH+Terms%5D%29+OR+%28Women%5BTitle%2FAbstract%5D+OR+Girls%5BTitle%2FAbstract%5D+OR+Girl%5BTitle%2FAbstract%5D+OR+Woman%5BTitle%2FAbstract%5D+OR+%22Women+Groups%22%5BTitle%2FAbstract%5D%29%29+OR+%28Female%5BMeSH+Terms%5D%29%29+OR+%28Female%5BTitle%2FAbstract%5D+OR+Females%5BTitle%2FAbstract%5D%29%29+AND+%28%28%28%28%28Domestic+Violence%5BMeSH+Terms%5D%29+OR+%28%22Domestic+Violence%22%5BTitle%2FAbstract%5D+OR+%22Violence%2C+Domestic%22%5BTitle%2FAbstract%5D+OR+%22Family+Violence%22%5BTitle%2FAbstract%5D+OR+%22Violence%2C+Family%22%5BTitle%2FAbstract%5D%29%29+OR+%28%22Violence+Against+Women%22%5BTitle%2FAbstract%5D%29%29+OR+%28%28Gender-Based+Violence%5BMeSH+Terms%5D%29+OR+%28%22Gender-Based+Violence%22%5BTitle%2FAbstract%5D+OR+%22Gender+Based+Violence%22%5BTitle%2FAbstract%5D+OR+%22Violence%2C+Gender-Based%22%5BTitle%2FAbstract%5D+OR+%22Dowry+Death%22%5BTitle%2FAbstract%5D+OR+%22Dowry+Deaths%22%5BTitle%2FAbstract%5D%29%29%29+OR+%28%28Intimate+Partner+Violence%5BMeSH+Terms%5D%29+OR+%28%22Intimate+Partner+Violence%22%5BTitle%2FAbstract%5D+OR+%22Partner+Violence%2C+Intimate%22%5BTitle%2FAbstract%5D+OR+%22Violence%2C+Intimate+Partner%22%5BTitle%2FAbstract%5D+OR+%22Intimate+Partner+Abuse%22%5BTitle%2FAbstract%5D+OR+%22Abuse%2C+Intimate+Partner%22%5BTitle%2FAbstract%5D+OR+%22Dating+Violence%22%5BTitle%2FAbstract%5D+OR+%22Violence%2C+Dating%22%5BTitle%2FAbstract%5D%29%29%29%29+AND+%28%28%28%22barriers+and+facilitators%22%5BTitle%2FAbstract%5D%29+OR+%28%22barrier+and+facilitator%22%5BTitle%2FAbstract%5D%29%29+OR+%28%28barriers%5BTitle%2FAbstract%5D+OR+barrier%5BTitle%2FAbstract%5D%29+AND+%28facilitators%5BTitle%2FAbstract%5D+OR+facilitator%5BTitle%2FAbstract%5D%29%29%29&sort=relevance&size=200&ac=no) | 12:45:09 |
| #21 |  |  | Search: **((((((Women[MeSH Terms]) OR (Women[Title/Abstract] OR Girls[Title/Abstract] OR Girl[Title/Abstract] OR Woman[Title/Abstract] OR "Women Groups"[Title/Abstract])) OR (Female[MeSH Terms])) OR (Female[Title/Abstract] OR Females[Title/Abstract])) AND (((((Domestic Violence[MeSH Terms]) OR ("Domestic Violence"[Title/Abstract] OR "Violence, Domestic"[Title/Abstract] OR "Family Violence"[Title/Abstract] OR "Violence, Family"[Title/Abstract])) OR ("Violence Against Women"[Title/Abstract])) OR ((Gender-Based Violence[MeSH Terms]) OR ("Gender-Based Violence"[Title/Abstract] OR "Gender Based Violence"[Title/Abstract] OR "Violence, Gender-Based"[Title/Abstract] OR "Dowry Death"[Title/Abstract] OR "Dowry Deaths"[Title/Abstract]))) OR ((Intimate Partner Violence[MeSH Terms]) OR ("Intimate Partner Violence"[Title/Abstract] OR "Partner Violence, Intimate"[Title/Abstract] OR "Violence, Intimate Partner"[Title/Abstract] OR "Intimate Partner Abuse"[Title/Abstract] OR "Abuse, Intimate Partner"[Title/Abstract] OR "Dating Violence"[Title/Abstract] OR "Violence, Dating"[Title/Abstract])))) AND ((("barriers and facilitators"[Title/Abstract]) OR ("barrier and facilitator"[Title/Abstract])) OR ((barriers[Title/Abstract] OR barrier[Title/Abstract]) AND (facilitators[Title/Abstract] OR facilitator[Title/Abstract])))) AND (((Health Personnel[MeSH Terms]) OR ("Health Personnel"[Title/Abstract] OR "Healthcare Workers"[Title/Abstract] OR "Healthcare Worker"[Title/Abstract] OR "Health Care Providers"[Title/Abstract] OR "Health Care Provider"[Title/Abstract] OR "Healthcare Providers"[Title/Abstract] OR "Healthcare Provider"[Title/Abstract] OR "Health Care Professionals"[Title/Abstract] OR "Health Care Professional"[Title/Abstract])) OR ("health care practitioner"[Title/Abstract] OR "health care professional"[Title/Abstract] OR "health care worker"[Title/Abstract] OR "health worker"[Title/Abstract] OR "healthcare personnel"[Title/Abstract] OR "healthcare practitioner"[Title/Abstract] OR "healthcare professional"[Title/Abstract] OR "healthcare worker"[Title/Abstract] OR "home health aides"[Title/Abstract] OR "public health officer"[Title/Abstract] OR "health care personnel"[Title/Abstract]))** | [53](https://pubmed.ncbi.nlm.nih.gov/?term=longqueryed40e4c567882322217e&ac=no&size=200&sort=relevance) | 12:43:06 |
| #22 |  |  | Search: **((((((Women[MeSH Terms]) OR (Women[Title/Abstract] OR Girls[Title/Abstract] OR Girl[Title/Abstract] OR Woman[Title/Abstract] OR "Women Groups"[Title/Abstract])) OR (Female[MeSH Terms])) OR (Female[Title/Abstract] OR Females[Title/Abstract])) AND (((((Domestic Violence[MeSH Terms]) OR ("Domestic Violence"[Title/Abstract] OR "Violence, Domestic"[Title/Abstract] OR "Family Violence"[Title/Abstract] OR "Violence, Family"[Title/Abstract])) OR ("Violence Against Women"[Title/Abstract])) OR ((Gender-Based Violence[MeSH Terms]) OR ("Gender-Based Violence"[Title/Abstract] OR "Gender Based Violence"[Title/Abstract] OR "Violence, Gender-Based"[Title/Abstract] OR "Dowry Death"[Title/Abstract] OR "Dowry Deaths"[Title/Abstract]))) OR ((Intimate Partner Violence[MeSH Terms]) OR ("Intimate Partner Violence"[Title/Abstract] OR "Partner Violence, Intimate"[Title/Abstract] OR "Violence, Intimate Partner"[Title/Abstract] OR "Intimate Partner Abuse"[Title/Abstract] OR "Abuse, Intimate Partner"[Title/Abstract] OR "Dating Violence"[Title/Abstract] OR "Violence, Dating"[Title/Abstract])))) AND ((("Evaluation of the Efficacy-Effectiveness of Interventions"[Title/Abstract]) OR ((Treatment Outcome[MeSH Terms]) OR ("Treatment Outcome"[Title/Abstract] OR "Treatment Efficacy"[Title/Abstract] OR "Clinical Efficacy"[Title/Abstract] OR "Rehabilitation Outcome"[Title/Abstract] OR "Clinical Effectiveness"[Title/Abstract] OR "Treatment Effectiveness"[Title/Abstract] OR "Patient-Relevant Outcome"[Title/Abstract] OR "Patient Relevant Outcome"[Title/Abstract] OR "Patient-Relevant Outcomes"[Title/Abstract]))) OR ("health care outcome and process assessment"[Title/Abstract] OR "medical futility"[Title/Abstract] OR "outcome AND process assessment (health care) "[Title/Abstract] OR "outcome management"[Title/Abstract] OR "patient outcome"[Title/Abstract] OR "therapeutic outcome"[Title/Abstract] OR "therapy outcome"[Title/Abstract]))) AND (((Health Personnel[MeSH Terms]) OR ("Health Personnel"[Title/Abstract] OR "Healthcare Workers"[Title/Abstract] OR "Healthcare Worker"[Title/Abstract] OR "Health Care Providers"[Title/Abstract] OR "Health Care Provider"[Title/Abstract] OR "Healthcare Providers"[Title/Abstract] OR "Healthcare Provider"[Title/Abstract] OR "Health Care Professionals"[Title/Abstract] OR "Health Care Professional"[Title/Abstract])) OR ("health care practitioner"[Title/Abstract] OR "health care professional"[Title/Abstract] OR "health care worker"[Title/Abstract] OR "health worker"[Title/Abstract] OR "healthcare personnel"[Title/Abstract] OR "healthcare practitioner"[Title/Abstract] OR "healthcare professional"[Title/Abstract] OR "healthcare worker"[Title/Abstract] OR "home health aides"[Title/Abstract] OR "public health officer"[Title/Abstract] OR "health care personnel"[Title/Abstract]))** | [24](https://pubmed.ncbi.nlm.nih.gov/?term=longquerye2c6ee11c05273c354f5&ac=no&size=200&sort=relevance) | 12:41:18 |
| #25 |  |  | Search: **((((((Women[MeSH Terms]) OR (Women[Title/Abstract] OR Girls[Title/Abstract] OR Girl[Title/Abstract] OR Woman[Title/Abstract] OR "Women Groups"[Title/Abstract])) OR (Female[MeSH Terms])) OR (Female[Title/Abstract] OR Females[Title/Abstract])) AND (((((Domestic Violence[MeSH Terms]) OR ("Domestic Violence"[Title/Abstract] OR "Violence, Domestic"[Title/Abstract] OR "Family Violence"[Title/Abstract] OR "Violence, Family"[Title/Abstract])) OR ("Violence Against Women"[Title/Abstract])) OR ((Gender-Based Violence[MeSH Terms]) OR ("Gender-Based Violence"[Title/Abstract] OR "Gender Based Violence"[Title/Abstract] OR "Violence, Gender-Based"[Title/Abstract] OR "Dowry Death"[Title/Abstract] OR "Dowry Deaths"[Title/Abstract]))) OR ((Intimate Partner Violence[MeSH Terms]) OR ("Intimate Partner Violence"[Title/Abstract] OR "Partner Violence, Intimate"[Title/Abstract] OR "Violence, Intimate Partner"[Title/Abstract] OR "Intimate Partner Abuse"[Title/Abstract] OR "Abuse, Intimate Partner"[Title/Abstract] OR "Dating Violence"[Title/Abstract] OR "Violence, Dating"[Title/Abstract])))) AND ((("Evaluation of the Efficacy-Effectiveness of Interventions"[Title/Abstract]) OR ((Treatment Outcome[MeSH Terms]) OR ("Treatment Outcome"[Title/Abstract] OR "Treatment Efficacy"[Title/Abstract] OR "Clinical Efficacy"[Title/Abstract] OR "Rehabilitation Outcome"[Title/Abstract] OR "Clinical Effectiveness"[Title/Abstract] OR "Treatment Effectiveness"[Title/Abstract] OR "Patient-Relevant Outcome"[Title/Abstract] OR "Patient Relevant Outcome"[Title/Abstract] OR "Patient-Relevant Outcomes"[Title/Abstract]))) OR ("health care outcome and process assessment"[Title/Abstract] OR "medical futility"[Title/Abstract] OR "outcome AND process assessment (health care) "[Title/Abstract] OR "outcome management"[Title/Abstract] OR "patient outcome"[Title/Abstract] OR "therapeutic outcome"[Title/Abstract] OR "therapy outcome"[Title/Abstract]))) AND (((Health Personnel[MeSH Terms]) OR ("Health Personnel"[Title/Abstract] OR "Healthcare Workers"[Title/Abstract] OR "Healthcare Worker"[Title/Abstract] OR "Health Care Providers"[Title/Abstract] OR "Health Care Provider"[Title/Abstract] OR "Healthcare Providers"[Title/Abstract] OR "Healthcare Provider"[Title/Abstract] OR "Health Care Professionals"[Title/Abstract] OR "Health Care Professional"[Title/Abstract])) OR ("health care practitioner"[Title/Abstract] OR "health care professional"[Title/Abstract] OR "health care worker"[Title/Abstract] OR "health worker"[Title/Abstract] OR "healthcare personnel"[Title/Abstract] OR "healthcare practitioner"[Title/Abstract] OR "healthcare professional"[Title/Abstract] OR "healthcare worker"[Title/Abstract] OR "home health aides"[Title/Abstract] OR "public health officer"[Title/Abstract] OR "health care personnel"[Title/Abstract]))** Filters: **Systematic Review** | 0 | 12:41:15 |
| #23 |  |  | Search: **((((((Women[MeSH Terms]) OR (Women[Title/Abstract] OR Girls[Title/Abstract] OR Girl[Title/Abstract] OR Woman[Title/Abstract] OR "Women Groups"[Title/Abstract])) OR (Female[MeSH Terms])) OR (Female[Title/Abstract] OR Females[Title/Abstract])) AND (((((Domestic Violence[MeSH Terms]) OR ("Domestic Violence"[Title/Abstract] OR "Violence, Domestic"[Title/Abstract] OR "Family Violence"[Title/Abstract] OR "Violence, Family"[Title/Abstract])) OR ("Violence Against Women"[Title/Abstract])) OR ((Gender-Based Violence[MeSH Terms]) OR ("Gender-Based Violence"[Title/Abstract] OR "Gender Based Violence"[Title/Abstract] OR "Violence, Gender-Based"[Title/Abstract] OR "Dowry Death"[Title/Abstract] OR "Dowry Deaths"[Title/Abstract]))) OR ((Intimate Partner Violence[MeSH Terms]) OR ("Intimate Partner Violence"[Title/Abstract] OR "Partner Violence, Intimate"[Title/Abstract] OR "Violence, Intimate Partner"[Title/Abstract] OR "Intimate Partner Abuse"[Title/Abstract] OR "Abuse, Intimate Partner"[Title/Abstract] OR "Dating Violence"[Title/Abstract] OR "Violence, Dating"[Title/Abstract])))) AND ((("barriers and facilitators"[Title/Abstract]) OR ("barrier and facilitator"[Title/Abstract])) OR ((barriers[Title/Abstract] OR barrier[Title/Abstract]) AND (facilitators[Title/Abstract] OR facilitator[Title/Abstract])))) AND (((Health Personnel[MeSH Terms]) OR ("Health Personnel"[Title/Abstract] OR "Healthcare Workers"[Title/Abstract] OR "Healthcare Worker"[Title/Abstract] OR "Health Care Providers"[Title/Abstract] OR "Health Care Provider"[Title/Abstract] OR "Healthcare Providers"[Title/Abstract] OR "Healthcare Provider"[Title/Abstract] OR "Health Care Professionals"[Title/Abstract] OR "Health Care Professional"[Title/Abstract])) OR ("health care practitioner"[Title/Abstract] OR "health care professional"[Title/Abstract] OR "health care worker"[Title/Abstract] OR "health worker"[Title/Abstract] OR "healthcare personnel"[Title/Abstract] OR "healthcare practitioner"[Title/Abstract] OR "healthcare professional"[Title/Abstract] OR "healthcare worker"[Title/Abstract] OR "home health aides"[Title/Abstract] OR "public health officer"[Title/Abstract] OR "health care personnel"[Title/Abstract]))** Filters: **Systematic Review** | [5](https://pubmed.ncbi.nlm.nih.gov/?term=longqueryed40e4c567882322217e&filter=pubt.systematicreview&ac=no&size=200&sort=relevance) | 12:39:21 |
| #24 |  |  | Search: **((((((Women[MeSH Terms]) OR (Women[Title/Abstract] OR Girls[Title/Abstract] OR Girl[Title/Abstract] OR Woman[Title/Abstract] OR "Women Groups"[Title/Abstract])) OR (Female[MeSH Terms])) OR (Female[Title/Abstract] OR Females[Title/Abstract])) AND (((((Domestic Violence[MeSH Terms]) OR ("Domestic Violence"[Title/Abstract] OR "Violence, Domestic"[Title/Abstract] OR "Family Violence"[Title/Abstract] OR "Violence, Family"[Title/Abstract])) OR ("Violence Against Women"[Title/Abstract])) OR ((Gender-Based Violence[MeSH Terms]) OR ("Gender-Based Violence"[Title/Abstract] OR "Gender Based Violence"[Title/Abstract] OR "Violence, Gender-Based"[Title/Abstract] OR "Dowry Death"[Title/Abstract] OR "Dowry Deaths"[Title/Abstract]))) OR ((Intimate Partner Violence[MeSH Terms]) OR ("Intimate Partner Violence"[Title/Abstract] OR "Partner Violence, Intimate"[Title/Abstract] OR "Violence, Intimate Partner"[Title/Abstract] OR "Intimate Partner Abuse"[Title/Abstract] OR "Abuse, Intimate Partner"[Title/Abstract] OR "Dating Violence"[Title/Abstract] OR "Violence, Dating"[Title/Abstract])))) AND ((("barriers and facilitators"[Title/Abstract]) OR ("barrier and facilitator"[Title/Abstract])) OR ((barriers[Title/Abstract] OR barrier[Title/Abstract]) AND (facilitators[Title/Abstract] OR facilitator[Title/Abstract])))) AND (((Health Personnel[MeSH Terms]) OR ("Health Personnel"[Title/Abstract] OR "Healthcare Workers"[Title/Abstract] OR "Healthcare Worker"[Title/Abstract] OR "Health Care Providers"[Title/Abstract] OR "Health Care Provider"[Title/Abstract] OR "Healthcare Providers"[Title/Abstract] OR "Healthcare Provider"[Title/Abstract] OR "Health Care Professionals"[Title/Abstract] OR "Health Care Professional"[Title/Abstract])) OR ("health care practitioner"[Title/Abstract] OR "health care professional"[Title/Abstract] OR "health care worker"[Title/Abstract] OR "health worker"[Title/Abstract] OR "healthcare personnel"[Title/Abstract] OR "healthcare practitioner"[Title/Abstract] OR "healthcare professional"[Title/Abstract] OR "healthcare worker"[Title/Abstract] OR "home health aides"[Title/Abstract] OR "public health officer"[Title/Abstract] OR "health care personnel"[Title/Abstract]))** Filters: **Meta-Analysis, Systematic Review** | [5](https://pubmed.ncbi.nlm.nih.gov/?term=longqueryed40e4c567882322217e&filter=pubt.meta-analysis&filter=pubt.systematicreview&ac=no&size=200&sort=relevance) | 12:38:59 |
| #20 |  |  | Search: **((Health Personnel[MeSH Terms]) OR ("Health Personnel"[Title/Abstract] OR "Healthcare Workers"[Title/Abstract] OR "Healthcare Worker"[Title/Abstract] OR "Health Care Providers"[Title/Abstract] OR "Health Care Provider"[Title/Abstract] OR "Healthcare Providers"[Title/Abstract] OR "Healthcare Provider"[Title/Abstract] OR "Health Care Professionals"[Title/Abstract] OR "Health Care Professional"[Title/Abstract])) OR ("health care practitioner"[Title/Abstract] OR "health care professional"[Title/Abstract] OR "health care worker"[Title/Abstract] OR "health worker"[Title/Abstract] OR "healthcare personnel"[Title/Abstract] OR "healthcare practitioner"[Title/Abstract] OR "healthcare professional"[Title/Abstract] OR "healthcare worker"[Title/Abstract] OR "home health aides"[Title/Abstract] OR "public health officer"[Title/Abstract] OR "health care personnel"[Title/Abstract])** | [776,311](https://pubmed.ncbi.nlm.nih.gov/?term=%28%28Health+Personnel%5BMeSH+Terms%5D%29+OR+%28%22Health+Personnel%22%5BTitle%2FAbstract%5D+OR+%22Healthcare+Workers%22%5BTitle%2FAbstract%5D+OR+%22Healthcare+Worker%22%5BTitle%2FAbstract%5D+OR+%22Health+Care+Providers%22%5BTitle%2FAbstract%5D+OR+%22Health+Care+Provider%22%5BTitle%2FAbstract%5D+OR+%22Healthcare+Providers%22%5BTitle%2FAbstract%5D+OR+%22Healthcare+Provider%22%5BTitle%2FAbstract%5D+OR+%22Health+Care+Professionals%22%5BTitle%2FAbstract%5D+OR+%22Health+Care+Professional%22%5BTitle%2FAbstract%5D%29%29+OR+%28%22health+care+practitioner%22%5BTitle%2FAbstract%5D+OR+%22health+care+professional%22%5BTitle%2FAbstract%5D+OR+%22health+care+worker%22%5BTitle%2FAbstract%5D+OR+%22health+worker%22%5BTitle%2FAbstract%5D+OR+%22healthcare+personnel%22%5BTitle%2FAbstract%5D+OR+%22healthcare+practitioner%22%5BTitle%2FAbstract%5D+OR+%22healthcare+professional%22%5BTitle%2FAbstract%5D+OR+%22healthcare+worker%22%5BTitle%2FAbstract%5D+OR+%22home+health+aides%22%5BTitle%2FAbstract%5D+OR+%22public+health+officer%22%5BTitle%2FAbstract%5D+OR+%22health+care+personnel%22%5BTitle%2FAbstract%5D%29&sort=relevance&size=200&ac=no) | 12:37:50 |
| #19 |  |  | Search: **"health care practitioner"[Title/Abstract] OR "health care professional"[Title/Abstract] OR "health care worker"[Title/Abstract] OR "health worker"[Title/Abstract] OR "healthcare personnel"[Title/Abstract] OR "healthcare practitioner"[Title/Abstract] OR "healthcare professional"[Title/Abstract] OR "healthcare worker"[Title/Abstract] OR "home health aides"[Title/Abstract] OR "public health officer"[Title/Abstract] OR "health care personnel"[Title/Abstract]** | [27,372](https://pubmed.ncbi.nlm.nih.gov/?term=%22health+care+practitioner%22%5BTitle%2FAbstract%5D+OR+%22health+care+professional%22%5BTitle%2FAbstract%5D+OR+%22health+care+worker%22%5BTitle%2FAbstract%5D+OR+%22health+worker%22%5BTitle%2FAbstract%5D+OR+%22healthcare+personnel%22%5BTitle%2FAbstract%5D+OR+%22healthcare+practitioner%22%5BTitle%2FAbstract%5D+OR+%22healthcare+professional%22%5BTitle%2FAbstract%5D+OR+%22healthcare+worker%22%5BTitle%2FAbstract%5D+OR+%22home+health+aides%22%5BTitle%2FAbstract%5D+OR+%22public+health+officer%22%5BTitle%2FAbstract%5D+OR+%22health+care+personnel%22%5BTitle%2FAbstract%5D&sort=relevance&size=200&ac=no) | 12:37:41 |
| #18 |  |  | Search: **(Health Personnel[MeSH Terms]) OR ("Health Personnel"[Title/Abstract] OR "Healthcare Workers"[Title/Abstract] OR "Healthcare Worker"[Title/Abstract] OR "Health Care Providers"[Title/Abstract] OR "Health Care Provider"[Title/Abstract] OR "Healthcare Providers"[Title/Abstract] OR "Healthcare Provider"[Title/Abstract] OR "Health Care Professionals"[Title/Abstract] OR "Health Care Professional"[Title/Abstract])** | [764,537](https://pubmed.ncbi.nlm.nih.gov/?term=%28Health+Personnel%5BMeSH+Terms%5D%29+OR+%28%22Health+Personnel%22%5BTitle%2FAbstract%5D+OR+%22Healthcare+Workers%22%5BTitle%2FAbstract%5D+OR+%22Healthcare+Worker%22%5BTitle%2FAbstract%5D+OR+%22Health+Care+Providers%22%5BTitle%2FAbstract%5D+OR+%22Health+Care+Provider%22%5BTitle%2FAbstract%5D+OR+%22Healthcare+Providers%22%5BTitle%2FAbstract%5D+OR+%22Healthcare+Provider%22%5BTitle%2FAbstract%5D+OR+%22Health+Care+Professionals%22%5BTitle%2FAbstract%5D+OR+%22Health+Care+Professional%22%5BTitle%2FAbstract%5D%29&sort=relevance&size=200&ac=no) | 12:37:35 |
| #17 |  |  | Search: **(((((Women[MeSH Terms]) OR (Women[Title/Abstract] OR Girls[Title/Abstract] OR Girl[Title/Abstract] OR Woman[Title/Abstract] OR "Women Groups"[Title/Abstract])) OR (Female[MeSH Terms])) OR (Female[Title/Abstract] OR Females[Title/Abstract])) AND (((((Domestic Violence[MeSH Terms]) OR ("Domestic Violence"[Title/Abstract] OR "Violence, Domestic"[Title/Abstract] OR "Family Violence"[Title/Abstract] OR "Violence, Family"[Title/Abstract])) OR ("Violence Against Women"[Title/Abstract])) OR ((Gender-Based Violence[MeSH Terms]) OR ("Gender-Based Violence"[Title/Abstract] OR "Gender Based Violence"[Title/Abstract] OR "Violence, Gender-Based"[Title/Abstract] OR "Dowry Death"[Title/Abstract] OR "Dowry Deaths"[Title/Abstract]))) OR ((Intimate Partner Violence[MeSH Terms]) OR ("Intimate Partner Violence"[Title/Abstract] OR "Partner Violence, Intimate"[Title/Abstract] OR "Violence, Intimate Partner"[Title/Abstract] OR "Intimate Partner Abuse"[Title/Abstract] OR "Abuse, Intimate Partner"[Title/Abstract] OR "Dating Violence"[Title/Abstract] OR "Violence, Dating"[Title/Abstract])))) AND ((("Evaluation of the Efficacy-Effectiveness of Interventions"[Title/Abstract]) OR ((Treatment Outcome[MeSH Terms]) OR ("Treatment Outcome"[Title/Abstract] OR "Treatment Efficacy"[Title/Abstract] OR "Clinical Efficacy"[Title/Abstract] OR "Rehabilitation Outcome"[Title/Abstract] OR "Clinical Effectiveness"[Title/Abstract] OR "Treatment Effectiveness"[Title/Abstract] OR "Patient-Relevant Outcome"[Title/Abstract] OR "Patient Relevant Outcome"[Title/Abstract] OR "Patient-Relevant Outcomes"[Title/Abstract]))) OR ("health care outcome and process assessment"[Title/Abstract] OR "medical futility"[Title/Abstract] OR "outcome AND process assessment (health care) "[Title/Abstract] OR "outcome management"[Title/Abstract] OR "patient outcome"[Title/Abstract] OR "therapeutic outcome"[Title/Abstract] OR "therapy outcome"[Title/Abstract]))** | [688](https://pubmed.ncbi.nlm.nih.gov/?term=longqueryfc2f832739be5dc652e7&sort=relevance&size=200&ac=no) | 12:31:43 |
| #16 |  |  | Search: **(("Evaluation of the Efficacy-Effectiveness of Interventions"[Title/Abstract]) OR ((Treatment Outcome[MeSH Terms]) OR ("Treatment Outcome"[Title/Abstract] OR "Treatment Efficacy"[Title/Abstract] OR "Clinical Efficacy"[Title/Abstract] OR "Rehabilitation Outcome"[Title/Abstract] OR "Clinical Effectiveness"[Title/Abstract] OR "Treatment Effectiveness"[Title/Abstract] OR "Patient-Relevant Outcome"[Title/Abstract] OR "Patient Relevant Outcome"[Title/Abstract] OR "Patient-Relevant Outcomes"[Title/Abstract]))) OR ("health care outcome and process assessment"[Title/Abstract] OR "medical futility"[Title/Abstract] OR "outcome AND process assessment (health care) "[Title/Abstract] OR "outcome management"[Title/Abstract] OR "patient outcome"[Title/Abstract] OR "therapeutic outcome"[Title/Abstract] OR "therapy outcome"[Title/Abstract])** | [1,432,218](https://pubmed.ncbi.nlm.nih.gov/?term=%28%28%22Evaluation+of+the+Efficacy-Effectiveness+of+Interventions%22%5BTitle%2FAbstract%5D%29+OR+%28%28Treatment+Outcome%5BMeSH+Terms%5D%29+OR+%28%22Treatment+Outcome%22%5BTitle%2FAbstract%5D+OR+%22Treatment+Efficacy%22%5BTitle%2FAbstract%5D+OR+%22Clinical+Efficacy%22%5BTitle%2FAbstract%5D+OR+%22Rehabilitation+Outcome%22%5BTitle%2FAbstract%5D+OR+%22Clinical+Effectiveness%22%5BTitle%2FAbstract%5D+OR+%22Treatment+Effectiveness%22%5BTitle%2FAbstract%5D+OR+%22Patient-Relevant+Outcome%22%5BTitle%2FAbstract%5D+OR+%22Patient+Relevant+Outcome%22%5BTitle%2FAbstract%5D+OR+%22Patient-Relevant+Outcomes%22%5BTitle%2FAbstract%5D%29%29%29+OR+%28%22health+care+outcome+and+process+assessment%22%5BTitle%2FAbstract%5D+OR+%22medical+futility%22%5BTitle%2FAbstract%5D+OR+%22outcome+AND+process+assessment+%28health+care%29+%22%5BTitle%2FAbstract%5D+OR+%22outcome+management%22%5BTitle%2FAbstract%5D+OR+%22patient+outcome%22%5BTitle%2FAbstract%5D+OR+%22therapeutic+outcome%22%5BTitle%2FAbstract%5D+OR+%22therapy+outcome%22%5BTitle%2FAbstract%5D%29&sort=relevance&size=200&ac=no) | 12:31:24 |
| #15 |  |  | Search: **"health care outcome and process assessment"[Title/Abstract] OR "medical futility"[Title/Abstract] OR "outcome AND process assessment (health care) "[Title/Abstract] OR "outcome management"[Title/Abstract] OR "patient outcome"[Title/Abstract] OR "therapeutic outcome"[Title/Abstract] OR "therapy outcome"[Title/Abstract]** | [28,114](https://pubmed.ncbi.nlm.nih.gov/?term=%22health+care+outcome+and+process+assessment%22%5BTitle%2FAbstract%5D+OR+%22medical+futility%22%5BTitle%2FAbstract%5D+OR+%22outcome+AND+process+assessment+%28health+care%29+%22%5BTitle%2FAbstract%5D+OR+%22outcome+management%22%5BTitle%2FAbstract%5D+OR+%22patient+outcome%22%5BTitle%2FAbstract%5D+OR+%22therapeutic+outcome%22%5BTitle%2FAbstract%5D+OR+%22therapy+outcome%22%5BTitle%2FAbstract%5D&sort=relevance&size=200&ac=no) | 12:31:10 |
| #14 |  |  | Search: **(Treatment Outcome[MeSH Terms]) OR ("Treatment Outcome"[Title/Abstract] OR "Treatment Efficacy"[Title/Abstract] OR "Clinical Efficacy"[Title/Abstract] OR "Rehabilitation Outcome"[Title/Abstract] OR "Clinical Effectiveness"[Title/Abstract] OR "Treatment Effectiveness"[Title/Abstract] OR "Patient-Relevant Outcome"[Title/Abstract] OR "Patient Relevant Outcome"[Title/Abstract] OR "Patient-Relevant Outcomes"[Title/Abstract])** | [1,410,776](https://pubmed.ncbi.nlm.nih.gov/?term=%28Treatment+Outcome%5BMeSH+Terms%5D%29+OR+%28%22Treatment+Outcome%22%5BTitle%2FAbstract%5D+OR+%22Treatment+Efficacy%22%5BTitle%2FAbstract%5D+OR+%22Clinical+Efficacy%22%5BTitle%2FAbstract%5D+OR+%22Rehabilitation+Outcome%22%5BTitle%2FAbstract%5D+OR+%22Clinical+Effectiveness%22%5BTitle%2FAbstract%5D+OR+%22Treatment+Effectiveness%22%5BTitle%2FAbstract%5D+OR+%22Patient-Relevant+Outcome%22%5BTitle%2FAbstract%5D+OR+%22Patient+Relevant+Outcome%22%5BTitle%2FAbstract%5D+OR+%22Patient-Relevant+Outcomes%22%5BTitle%2FAbstract%5D%29&sort=relevance&size=200&ac=no) | 12:28:29 |
| #13 |  |  | Search: **"Evaluation of the Efficacy-Effectiveness of Interventions"[Title/Abstract]** | [4](https://pubmed.ncbi.nlm.nih.gov/?term=%22Evaluation+of+the+Efficacy-Effectiveness+of+Interventions%22%5BTitle%2FAbstract%5D&sort=relevance&size=200&ac=no) | 12:27:29 |
| #11 |  |  | Search: **(("barriers and facilitators"[Title/Abstract]) OR ("barrier and facilitator"[Title/Abstract])) OR ((barriers[Title/Abstract] OR barrier[Title/Abstract]) AND (facilitators[Title/Abstract] OR facilitator[Title/Abstract]))** | [25,253](https://pubmed.ncbi.nlm.nih.gov/?term=%28%28%22barriers+and+facilitators%22%5BTitle%2FAbstract%5D%29+OR+%28%22barrier+and+facilitator%22%5BTitle%2FAbstract%5D%29%29+OR+%28%28barriers%5BTitle%2FAbstract%5D+OR+barrier%5BTitle%2FAbstract%5D%29+AND+%28facilitators%5BTitle%2FAbstract%5D+OR+facilitator%5BTitle%2FAbstract%5D%29%29&sort=relevance&size=200&ac=no) | 12:26:46 |
| #10 |  |  | Search: **(barriers[Title/Abstract] OR barrier[Title/Abstract]) AND (facilitators[Title/Abstract] OR facilitator[Title/Abstract])** | [25,253](https://pubmed.ncbi.nlm.nih.gov/?term=%28barriers%5BTitle%2FAbstract%5D+OR+barrier%5BTitle%2FAbstract%5D%29+AND+%28facilitators%5BTitle%2FAbstract%5D+OR+facilitator%5BTitle%2FAbstract%5D%29&sort=relevance&size=200&ac=no) | 12:26:35 |
| #9 |  |  | Search: **"barrier and facilitator"[Title/Abstract]** | [125](https://pubmed.ncbi.nlm.nih.gov/?term=%22barrier+and+facilitator%22%5BTitle%2FAbstract%5D&sort=relevance&size=200&ac=no) | 12:25:52 |
| #8 |  |  | Search: **"barriers and facilitators"[Title/Abstract]** | [14,019](https://pubmed.ncbi.nlm.nih.gov/?term=%22barriers+and+facilitators%22%5BTitle%2FAbstract%5D&sort=relevance&size=200&ac=no) | 12:25:45 |
| #7 |  |  | Search: **((((Women[MeSH Terms]) OR (Women[Title/Abstract] OR Girls[Title/Abstract] OR Girl[Title/Abstract] OR Woman[Title/Abstract] OR "Women Groups"[Title/Abstract])) OR (Female[MeSH Terms])) OR (Female[Title/Abstract] OR Females[Title/Abstract])) AND (((((Domestic Violence[MeSH Terms]) OR ("Domestic Violence"[Title/Abstract] OR "Violence, Domestic"[Title/Abstract] OR "Family Violence"[Title/Abstract] OR "Violence, Family"[Title/Abstract])) OR ("Violence Against Women"[Title/Abstract])) OR ((Gender-Based Violence[MeSH Terms]) OR ("Gender-Based Violence"[Title/Abstract] OR "Gender Based Violence"[Title/Abstract] OR "Violence, Gender-Based"[Title/Abstract] OR "Dowry Death"[Title/Abstract] OR "Dowry Deaths"[Title/Abstract]))) OR ((Intimate Partner Violence[MeSH Terms]) OR ("Intimate Partner Violence"[Title/Abstract] OR "Partner Violence, Intimate"[Title/Abstract] OR "Violence, Intimate Partner"[Title/Abstract] OR "Intimate Partner Abuse"[Title/Abstract] OR "Abuse, Intimate Partner"[Title/Abstract] OR "Dating Violence"[Title/Abstract] OR "Violence, Dating"[Title/Abstract])))** | [47,466](https://pubmed.ncbi.nlm.nih.gov/?term=%28%28%28%28Women%5BMeSH+Terms%5D%29+OR+%28Women%5BTitle%2FAbstract%5D+OR+Girls%5BTitle%2FAbstract%5D+OR+Girl%5BTitle%2FAbstract%5D+OR+Woman%5BTitle%2FAbstract%5D+OR+%22Women+Groups%22%5BTitle%2FAbstract%5D%29%29+OR+%28Female%5BMeSH+Terms%5D%29%29+OR+%28Female%5BTitle%2FAbstract%5D+OR+Females%5BTitle%2FAbstract%5D%29%29+AND+%28%28%28%28%28Domestic+Violence%5BMeSH+Terms%5D%29+OR+%28%22Domestic+Violence%22%5BTitle%2FAbstract%5D+OR+%22Violence%2C+Domestic%22%5BTitle%2FAbstract%5D+OR+%22Family+Violence%22%5BTitle%2FAbstract%5D+OR+%22Violence%2C+Family%22%5BTitle%2FAbstract%5D%29%29+OR+%28%22Violence+Against+Women%22%5BTitle%2FAbstract%5D%29%29+OR+%28%28Gender-Based+Violence%5BMeSH+Terms%5D%29+OR+%28%22Gender-Based+Violence%22%5BTitle%2FAbstract%5D+OR+%22Gender+Based+Violence%22%5BTitle%2FAbstract%5D+OR+%22Violence%2C+Gender-Based%22%5BTitle%2FAbstract%5D+OR+%22Dowry+Death%22%5BTitle%2FAbstract%5D+OR+%22Dowry+Deaths%22%5BTitle%2FAbstract%5D%29%29%29+OR+%28%28Intimate+Partner+Violence%5BMeSH+Terms%5D%29+OR+%28%22Intimate+Partner+Violence%22%5BTitle%2FAbstract%5D+OR+%22Partner+Violence%2C+Intimate%22%5BTitle%2FAbstract%5D+OR+%22Violence%2C+Intimate+Partner%22%5BTitle%2FAbstract%5D+OR+%22Intimate+Partner+Abuse%22%5BTitle%2FAbstract%5D+OR+%22Abuse%2C+Intimate+Partner%22%5BTitle%2FAbstract%5D+OR+%22Dating+Violence%22%5BTitle%2FAbstract%5D+OR+%22Violence%2C+Dating%22%5BTitle%2FAbstract%5D%29%29%29&sort=relevance&size=200&ac=no) | 12:25:33 |
| #6 |  |  | Search: **((((Domestic Violence[MeSH Terms]) OR ("Domestic Violence"[Title/Abstract] OR "Violence, Domestic"[Title/Abstract] OR "Family Violence"[Title/Abstract] OR "Violence, Family"[Title/Abstract])) OR ("Violence Against Women"[Title/Abstract])) OR ((Gender-Based Violence[MeSH Terms]) OR ("Gender-Based Violence"[Title/Abstract] OR "Gender Based Violence"[Title/Abstract] OR "Violence, Gender-Based"[Title/Abstract] OR "Dowry Death"[Title/Abstract] OR "Dowry Deaths"[Title/Abstract]))) OR ((Intimate Partner Violence[MeSH Terms]) OR ("Intimate Partner Violence"[Title/Abstract] OR "Partner Violence, Intimate"[Title/Abstract] OR "Violence, Intimate Partner"[Title/Abstract] OR "Intimate Partner Abuse"[Title/Abstract] OR "Abuse, Intimate Partner"[Title/Abstract] OR "Dating Violence"[Title/Abstract] OR "Violence, Dating"[Title/Abstract]))** | [68,095](https://pubmed.ncbi.nlm.nih.gov/?term=%28%28%28%28Domestic+Violence%5BMeSH+Terms%5D%29+OR+%28%22Domestic+Violence%22%5BTitle%2FAbstract%5D+OR+%22Violence%2C+Domestic%22%5BTitle%2FAbstract%5D+OR+%22Family+Violence%22%5BTitle%2FAbstract%5D+OR+%22Violence%2C+Family%22%5BTitle%2FAbstract%5D%29%29+OR+%28%22Violence+Against+Women%22%5BTitle%2FAbstract%5D%29%29+OR+%28%28Gender-Based+Violence%5BMeSH+Terms%5D%29+OR+%28%22Gender-Based+Violence%22%5BTitle%2FAbstract%5D+OR+%22Gender+Based+Violence%22%5BTitle%2FAbstract%5D+OR+%22Violence%2C+Gender-Based%22%5BTitle%2FAbstract%5D+OR+%22Dowry+Death%22%5BTitle%2FAbstract%5D+OR+%22Dowry+Deaths%22%5BTitle%2FAbstract%5D%29%29%29+OR+%28%28Intimate+Partner+Violence%5BMeSH+Terms%5D%29+OR+%28%22Intimate+Partner+Violence%22%5BTitle%2FAbstract%5D+OR+%22Partner+Violence%2C+Intimate%22%5BTitle%2FAbstract%5D+OR+%22Violence%2C+Intimate+Partner%22%5BTitle%2FAbstract%5D+OR+%22Intimate+Partner+Abuse%22%5BTitle%2FAbstract%5D+OR+%22Abuse%2C+Intimate+Partner%22%5BTitle%2FAbstract%5D+OR+%22Dating+Violence%22%5BTitle%2FAbstract%5D+OR+%22Violence%2C+Dating%22%5BTitle%2FAbstract%5D%29%29&sort=relevance&size=200&ac=no) | 12:25:19 |
| #5 |  |  | Search: **(Intimate Partner Violence[MeSH Terms]) OR ("Intimate Partner Violence"[Title/Abstract] OR "Partner Violence, Intimate"[Title/Abstract] OR "Violence, Intimate Partner"[Title/Abstract] OR "Intimate Partner Abuse"[Title/Abstract] OR "Abuse, Intimate Partner"[Title/Abstract] OR "Dating Violence"[Title/Abstract] OR "Violence, Dating"[Title/Abstract])** | [19,488](https://pubmed.ncbi.nlm.nih.gov/?term=%28Intimate+Partner+Violence%5BMeSH+Terms%5D%29+OR+%28%22Intimate+Partner+Violence%22%5BTitle%2FAbstract%5D+OR+%22Partner+Violence%2C+Intimate%22%5BTitle%2FAbstract%5D+OR+%22Violence%2C+Intimate+Partner%22%5BTitle%2FAbstract%5D+OR+%22Intimate+Partner+Abuse%22%5BTitle%2FAbstract%5D+OR+%22Abuse%2C+Intimate+Partner%22%5BTitle%2FAbstract%5D+OR+%22Dating+Violence%22%5BTitle%2FAbstract%5D+OR+%22Violence%2C+Dating%22%5BTitle%2FAbstract%5D%29&sort=relevance&size=200&ac=no) | 12:25:05 |
| #4 |  |  | Search: **(Gender-Based Violence[MeSH Terms]) OR ("Gender-Based Violence"[Title/Abstract] OR "Gender Based Violence"[Title/Abstract] OR "Violence, Gender-Based"[Title/Abstract] OR "Dowry Death"[Title/Abstract] OR "Dowry Deaths"[Title/Abstract])** | [2,509](https://pubmed.ncbi.nlm.nih.gov/?term=%28Gender-Based+Violence%5BMeSH+Terms%5D%29+OR+%28%22Gender-Based+Violence%22%5BTitle%2FAbstract%5D+OR+%22Gender+Based+Violence%22%5BTitle%2FAbstract%5D+OR+%22Violence%2C+Gender-Based%22%5BTitle%2FAbstract%5D+OR+%22Dowry+Death%22%5BTitle%2FAbstract%5D+OR+%22Dowry+Deaths%22%5BTitle%2FAbstract%5D%29&sort=relevance&size=200&ac=no) | 12:24:45 |
| #3 |  |  | Search: **"Violence Against Women"[Title/Abstract]** | [3,911](https://pubmed.ncbi.nlm.nih.gov/?term=%22Violence+Against+Women%22%5BTitle%2FAbstract%5D&sort=relevance&size=200&ac=no) | 12:24:26 |
| #2 |  |  | Search: **(Domestic Violence[MeSH Terms]) OR ("Domestic Violence"[Title/Abstract] OR "Violence, Domestic"[Title/Abstract] OR "Family Violence"[Title/Abstract] OR "Violence, Family"[Title/Abstract])** | [57,102](https://pubmed.ncbi.nlm.nih.gov/?term=%28Domestic+Violence%5BMeSH+Terms%5D%29+OR+%28%22Domestic+Violence%22%5BTitle%2FAbstract%5D+OR+%22Violence%2C+Domestic%22%5BTitle%2FAbstract%5D+OR+%22Family+Violence%22%5BTitle%2FAbstract%5D+OR+%22Violence%2C+Family%22%5BTitle%2FAbstract%5D%29&sort=relevance&size=200&ac=no) | 12:24:14 |
| #1 |  |  | Search: **(((Women[MeSH Terms]) OR (Women[Title/Abstract] OR Girls[Title/Abstract] OR Girl[Title/Abstract] OR Woman[Title/Abstract] OR "Women Groups"[Title/Abstract])) OR (Female[MeSH Terms])) OR (Female[Title/Abstract] OR Females[Title/Abstract])** | [10,543,759](https://pubmed.ncbi.nlm.nih.gov/?term=%28%28%28Women%5BMeSH+Terms%5D%29+OR+%28Women%5BTitle%2FAbstract%5D+OR+Girls%5BTitle%2FAbstract%5D+OR+Girl%5BTitle%2FAbstract%5D+OR+Woman%5BTitle%2FAbstract%5D+OR+%22Women+Groups%22%5BTitle%2FAbstract%5D%29%29+OR+%28Female%5BMeSH+Terms%5D%29%29+OR+%28Female%5BTitle%2FAbstract%5D+OR+Females%5BTitle%2FAbstract%5D%29&sort=relevance&size=200&ac=no) | 12:23:56 |

Showing 1 to 25 of 25 entries

**Table S2.** JBI Critical Appraisal Checklist for Systematic Reviews and Research Syntheses

| **#** | **Author/year/Country** | **Title** | **Q1** | **Q2** | **Q3** | **Q4** | **Q5** | **Q6** | **Q7** | **Q8** | **Q9** | **Q10** | **Q11** | **Score** |
| --- | --- | --- | --- | --- | --- | --- | --- | --- | --- | --- | --- | --- | --- | --- |
| 1 | Saletti-Cuesta L, et al., 2018  Argentina and Spain | Opinions and Experiences of Primary Healthcare Providers Regarding Violence against Women: a Systematic Review of Qualitative Studies | Y | Y | Y | Y | Y | Y | Y | Y | N/A | Y | Y | 10Y 1N/A |
| 2 | Bundock K, et al., 2020  UK | Adolescents' Help-Seeking Behavior and Intentions Following Adolescent Dating Violence: A Systematic Review | Y | Y | Y | Y | Y | U | U | U | N/A | Y | Y | 7Y 3U 1N/A |
| 3 | Duchesne E et al., 2023  Canada | Patient and Provider Emergency Care Experiences Related to Intimate Partner Violence: A Systematic Review of the Existing Evidence | Y | Y | Y | Y | Y | Y | U | U | N/A | Y | Y | 8Y 2U 1N/A |
| 4 | Hulley J, et al., 2023  UK | Intimate Partner Violence and Barriers to Help-Seeking Among Black, Asian, Minority Ethnic and Immigrant Women: A Qualitative Metasynthesis of Global Research | Y | Y | Y | Y | Y | Y | U | Y | Y | Y | Y | 10Y 1U |
| 5 | Ravi KE, et al., 2022  USA | Facilitators of Formal Help-Seeking for Adult Survivors of IPV in the United States: A Systematic Review | Y | Y | Y | Y | N | N | U | U | N/A | Y | Y | 6Y 2N 2U 1N/A |
| 6 | Lu C, et al., 2023  Australia | Identifying the barriers faced by obstetricians and registrars in screening or enquiry of intimate partner violence in pregnancy: A systematic review of the primary evidence | Y | Y | Y | Y | Y | Y | Y | U | Y | U | U | 8Y 3U |
| 7 | Femi-Ajao O, et al., 2020  UK | A qualitative systematic review of published work on disclosure and help-seeking for domestic violence and abuse among women from ethnic minority populations in the UK | Y | Y | Y | Y | Y | Y | Y | Y | N/A | Y | Y | 10Y 1N/A |
| 8 | Colombini M, et al., 2017 UK | Barriers and Facilitators to Integrating Health Service Responses to Intimate Partner Violence in Low- and Middle-Income Countries: A Comparative Health Systems and Service Analysis | Y | Y | Y | Y | Y | U | Y | Y | N/A | Y | Y | 9Y 1U 1N/A |
| 9 | Brown SJ, et al., 2022 Australia and UK | Survivor, family and professional experiences of psychosocial interventions for sexual abuse and violence: a qualitative evidence synthesis | Y | Y | Y | Y | Y | Y | Y | Y | Y | Y | Y | 11Y |
| 10 | Sultana R, et al., 2023  UK | A Systematic Review and Meta-Synthesis of Barriers and Facilitators of Help-Seeking Behaviors in South Asian Women Living in High-Income Countries who Have Experienced Domestic Violence: Perception of Domestic Violence Survivors and Service Providers. | Y | Y | Y | Y | Y | Y | Y | Y | N/A | Y | Y | 10Y 1N/A |
| 11 | Heron RL, Eisma MC, et al. 2021 USA and The Netherlands | Barriers and facilitators of disclosing domestic violence to the healthcare service: A systematic review of qualitative research. | Y | Y | Y | N | Y | Y | Y | U | N/A | Y | Y | 8Y 1N 1U 1N/A |

Y=Yes/N=Not/U=Unclear/NA=Not applicable

JBI Critical Appraisal Checklist for Systematic Reviews and Research Syntheses [11] questions

1. Is the review question clearly and explicitly stated?
2. Were the inclusion criteria appropriate for the review question?
3. Was the search strategy appropriate?
4. Were the sources and resources used to search for studies adequate?
5. Were the criteria for appraising studies appropriate?
6. Was critical appraisal conducted by two or more reviewers independently?
7. Were there methods to minimize errors in data extraction?
8. Were the methods used to combine studies appropriate?
9. Was the likelihood of publication bias assessed?
10. Were recommendations for policy and/or practice supported by the reported data?
11. Were the specific directives for new research appropriate?

**Preferred Reporting Items for Systematic reviews and Meta-Analyses extension for Scoping Reviews (PRISMA-ScR) Checklist**

| **SECTION** | **ITEM** | **PRISMA-ScR CHECKLIST ITEM** | **REPORTED ON PAGE #** |
| --- | --- | --- | --- |
| **TITLE** | | | |
| Title | 1 | Identify the report as a scoping review. | N/A |
| **ABSTRACT** | | | |
| Structured summary | 2 | Provide a structured summary that includes (as applicable): background, objectives, eligibility criteria, sources of evidence, charting methods, results, and conclusions that relate to the review questions and objectives. | p.1-2 |
| **INTRODUCTION** | | | |
| Rationale | 3 | Describe the rationale for the review in the context of what is already known. Explain why the review questions/objectives lend themselves to a scoping review approach. | p.3-5 |
| Objectives | 4 | Provide an explicit statement of the questions and objectives being addressed with reference to their key elements (e.g., population or participants, concepts, and context) or other relevant key elements used to conceptualize the review questions and/or objectives. | p.5 |
| **METHODS** | | | |
| Protocol and registration | 5 | Indicate whether a review protocol exists; state if and where it can be accessed (e.g., a Web address); and if available, provide registration information, including the registration number. | p. 6 |
| Eligibility criteria | 6 | Specify characteristics of the sources of evidence used as eligibility criteria (e.g., years considered, language, and publication status), and provide a rationale. | p.6 |
| Information sources* | 7 | Describe all information sources in the search (e.g., databases with dates of coverage and contact with authors to identify additional sources), as well as the date the most recent search was executed. | p.6-7 |
| Search | 8 | Present the full electronic search strategy for at least 1 database, including any limits used, such that it could be repeated. | Table S1 |
| Selection of sources of evidence† | 9 | State the process for selecting sources of evidence (i.e., screening and eligibility) included in the scoping review. | p.7 |
| Data charting process‡ | 10 | Describe the methods of charting data from the included sources of evidence (e.g., calibrated forms or forms that have been tested by the team before their use, and whether data charting was done independently or in duplicate) and any processes for obtaining and confirming data from investigators. | p.7-8 |
| Data items | 11 | List and define all variables for which data were sought and any assumptions and simplifications made. | p.7 |
| Critical appraisal of individual sources of evidence§ | 12 | If done, provide a rationale for conducting a critical appraisal of included sources of evidence; describe the methods used and how this information was used in any data synthesis (if appropriate). | p.8 |
| Synthesis of results | 13 | Describe the methods of handling and summarizing the data that were charted. | p.8 |
| **RESULTS** | | | |
| Selection of sources of evidence | 14 | Give numbers of sources of evidence screened, assessed for eligibility, and included in the review, with reasons for exclusions at each stage, ideally using a flow diagram. | Figure 1 |
| Characteristics of sources of evidence | 15 | For each source of evidence, present characteristics for which data were charted and provide the citations. | p.9-10 |
| Critical appraisal within sources of evidence | 16 | If done, present data on critical appraisal of included sources of evidence (see item 12). | Table S2 |
| Results of individual sources of evidence | 17 | For each included source of evidence, present the relevant data that were charted that relate to the review questions and objectives. | Table 1 and Table 2 |
| Synthesis of results | 18 | Summarize and/or present the charting results as they relate to the review questions and objectives. | p.11-20 |
| **DISCUSSION** | | | |
| Summary of evidence | 19 | Summarize the main results (including an overview of concepts, themes, and types of evidence available), link to the review questions and objectives, and consider the relevance to key groups. | p.20-24 |
| Limitations | 20 | Discuss the limitations of the scoping review process. | p.24 |
| Conclusions | 21 | Provide a general interpretation of the results with respect to the review questions and objectives, as well as potential implications and/or next steps. | p.25 |
| **FUNDING** | | | |
| Funding | 22 | Describe sources of funding for the included sources of evidence, as well as sources of funding for the scoping review. Describe the role of the funders of the scoping review. | p.25-26 |

JBI = Joanna Briggs Institute; PRISMA-ScR = Preferred Reporting Items for Systematic reviews and Meta-Analyses extension for Scoping Reviews.

* Where *sources of evidence* (see second footnote) are compiled from, such as bibliographic databases, social media platforms, and Web sites.

† A more inclusive/heterogeneous term used to account for the different types of evidence or data sources (e.g., quantitative and/or qualitative research, expert opinion, and policy documents) that may be eligible in a scoping review as opposed to only studies. This is not to be confused with *information sources* (see first footnote).

‡ The frameworks by Arksey and O’Malley (6) and Levac and colleagues (7) and the JBI guidance (4, 5) refer to the process of data extraction in a scoping review as data charting*.*

§ The process of systematically examining research evidence to assess its validity, results, and relevance before using it to inform a decision. This term is used for items 12 and 19 instead of "risk of bias" (which is more applicable to systematic reviews of interventions) to include and acknowledge the various sources of evidence that may be used in a scoping review (e.g., quantitative and/or qualitative research, expert opinion, and policy document).

*From:* Tricco AC, Lillie E, Zarin W, O'Brien KK, Colquhoun H, Levac D, et al. PRISMA Extension for Scoping Reviews (PRISMAScR): Checklist and Explanation. Ann Intern Med. 2018;169:467–473. [doi: 10.7326/M18-0850](http://annals.org/aim/fullarticle/2700389/prisma-extension-scoping-reviews-prisma-scr-checklist-explanation).
